# Development of personalized non-invasive ventilation masks for critically ill children: a bench study

**DOI:** 10.1186/s40635-024-00607-w

**Published:** 2024-03-01

**Authors:** Rosemijne R. W. P. Pigmans, Rozalinde Klein-Blommert, Monica C. van Gestel, Dick G. Markhorst, Peter Hammond, Pim Boomsma, Tim Daams, Julia M. A. de Jong, Paul M. Heeman, Job B. M. van Woensel, Coen D. Dijkman, Reinout A. Bem

**Affiliations:** 1 Pediatric Intensive Care Unit, Emma Children’s Hospital, Amsterdam UMC, Location AMC, University of Amsterdam, Meibergdreef 9, 1105 AZ Amsterdam, The Netherlands; 2 Amsterdam Reproduction and Development Research Institute, Amsterdam, The Netherlands; 3 Nuffield Department of Women’s and Reproductive Health, University of Oxford, Oxford, UK; 4 Big Data Institute, Old Road Campus, University of Oxford, Oxford, UK; 5 Department for Medical Innovation and Development, Amsterdam University Medical Centers, Amsterdam, The Netherlands

**Keywords:** Face mask, Customization, 3D printing, Acute respiratory failure, Non-invasive respiratory support, Pediatric intensive care unit

## Abstract

**Background:**

Obtaining a properly fitting non-invasive ventilation (NIV) mask to treat acute respiratory failure is a major challenge, especially in young children and patients with craniofacial abnormalities. Personalization of NIV masks holds promise to improve pediatric NIV efficiency. As current customization methods are relatively time consuming, this study aimed to test the air leak and surface pressure performance of personalized oronasal face masks using 3D printed soft materials. Personalized masks of three different biocompatible materials (silicone and photopolymer resin) were developed and tested on three head models of young children with abnormal facial features during preclinical bench simulation of pediatric NIV. Air leak percentages and facial surface pressures were measured and compared for each mask.

**Results:**

Personalized NIV masks could be successfully produced in under 12 h in a semi-automated 3D production process. During NIV simulation, overall air leak performance and applied surface pressures were acceptable, with leak percentages under 30% and average surface pressure values mostly remaining under normal capillary pressure. There was a small advantage of the masks produced with soft photopolymer resin material.

**Conclusion:**

This first, proof-of-concept bench study simulating NIV in children with abnormal facial features, showed that it is possible to obtain biocompatible, personalized oronasal masks with acceptable air leak and facial surface pressure performance using a relatively short, and semi-automated production process. Further research into the clinical value and possibilities for application of personalized NIV masks in critically ill children is needed.

**Supplementary Information:**

The online version contains supplementary material available at 10.1186/s40635-024-00607-w.

## Background

Non-invasive ventilation (NIV) can be a valuable treatment in critically ill children with acute respiratory failure admitted to the pediatric intensive care unit (PICU) [1, 2]. A recent worldwide study reported that almost 1 out of every 4 children with pediatric acute respiratory distress syndrome currently receives this treatment [3]. One of the most important challenges in treating children with NIV is to obtain a properly fitting mask [2, 4–9]. A suboptimal seal of the NIV mask results in increased air leak, patient discomfort and the development of facial skin pressure injuries [9, 10]. These factors are associated with patient–ventilator asynchrony and reduced tolerance, contributing to NIV failure [8, 9, 11–13]. This is relevant as several large studies report NIV failure rates in the PICU to range from 25 to 53% [3, 14]. Young children are particularly exposed to ill-fitted NIV interfaces, as a result of the limited size ranges of commercially available masks. In addition, syndromic craniofacial malformations in children (a common problem in patients admitted to the PICU) might further complicate mask fitting [8, 11, 12]. Therefore, personalization of NIV masks addressing the specific facial features of the patient is believed to be a promising future approach to improve pediatric NIV efficiency [4–9].

There have been several previous explorations in the field of mask personalization [9, 15–18]. Biocompatible soft materials, like silicon, which has a cushioning effect to minimize pressure on the face and improve seal compared to hard materials, were investigated using molding techniques to produce a mask [9, 15, 16]. However, such strategies are labor and time consuming, which may limit their usefulness in the setting of acute respiratory failure. More novel, state-of-the-art techniques, such as 3D printing of soft materials may be promising to provide a more rapid, personalized NIV mask production method [6, 17, 18]. Yet, medical device research aimed at establishing 3D printed personalized masks is still in the early stage. For example, Willox et al. [18] tested 3D printed polyamide oronasal masks in three healthy adult volunteers, and found reduced air leak as compared to conventional NIV masks, but also expressed the need to test softer materials. Borras-Novell et al. [17] tested a 3D printed, softer, silicone nasal mask in a single neonatal case, and found reduced air leak percentages. Nevertheless, before pilot and further clinical studies in children with acute respiratory failure can be attempted, further exploration of the production process, and performance of designs and materials in a preclinical setting is needed.

The aim of this study was to explore 3D printed personalized oronasal NIV face masks produced with different biocompatible soft materials, and test their air leak and facial surface pressure performance in preclinical bench simulation of pediatric NIV. For this purpose, we developed head models of young children with abnormal facial features for a proof-of-concept study.

## Methods

### Development of pediatric head models

Three different head models were developed, based on the facial 3D scan of three children (aged 3–4 years) with a syndrome with accompanying characteristic facial features: Down syndrome (DS, Trisomy 21), cardiofaciocutaneous syndrome (CFCS) and velocardiofacial syndrome (VCFS). These children were selected based on evident facial abnormalities as part of their syndrome, to provide for a proof-of-concept model to test our personalized mask design and materials. The 3D images were obtained with approval of the Joint Research and Ethics Committee of the Eastman Dental Institute/Eastman Dental Hospital Ethics Committee (JREC 00/EO42). The scans were digitally transformed into solid heads by merging the scans with the back of a standard head in Autodesk Meshmixer (version 3.5.474, Autodesk, San Rafael, CA, USA).

**Fig. 1:**
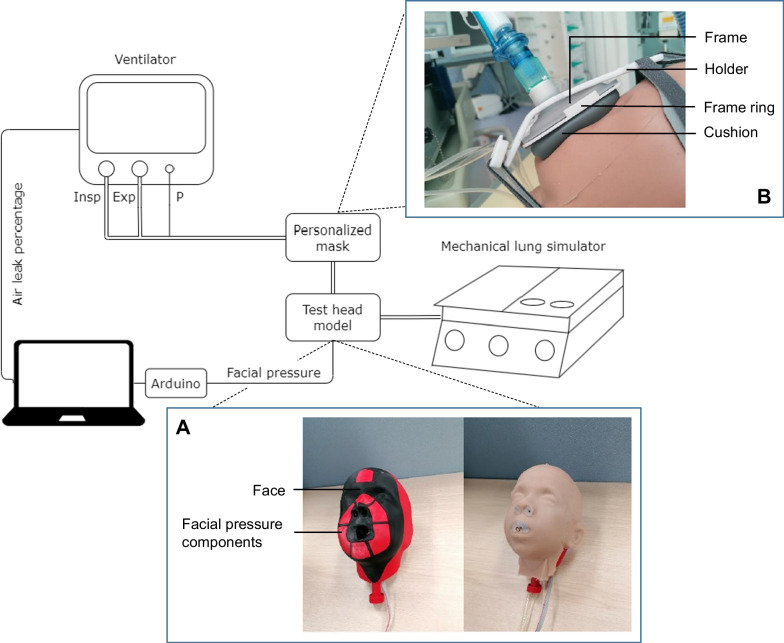
A schematic overview of the bench test setup for pediatric non-invasive ventilation simulation with details on the test head model (**A**) and the personalized mask (**B**). The personalized mask consist of a frame, frame ring, holder and a personalized cushion. The mask is placed on the accompanying test head model, which contains facial pressure components that follow the outline of the ventilation mask. The pressure sensors underneath the components are connected to the laptop through a microcontroller (Arduino). The test head model has a 3-mm silicon layer to create an airtight connection to the mechanical lung simulator. The mask is connected to the ventilator, which directly provides information on the inspiration (insp) and expiration (exp) volumes, flows and pressures (P) to the laptop for data collection

The pediatric head models were developed in Autodesk Inventor (Autodesk, USA). An example of a final head model as produced is shown in Fig. 1A. All three test head models are shown in Additional file 1: Figure S1. All head models were composed of four solid, 3D printed parts (ASA, Fortus 450mc Industrial FDM printer, Stratasys, Israel): the face, middle and back of the head, and the facial pressure components. Six capacitive force sensors (4.5N, calibrated, Singletact, PSS UK Limited, UK) were placed under the pressure components on a flat surface following the contours of NIV oronasal masks. The measured force was converted to pressure by dividing the output by the surface area of the corresponding pressure component. The regions for pressure measurements include the nose bridge, both sides of the nose, both cheeks and the chin. They, respectively, enclose the nasion, nasal ala left and right, chelion left and right and the sulcus inferior facial landmarks. The electronics of the sensors were placed in the back part of the head. The sensors were coupled and connected to a microcontroller (Arduino Uno, Arduino, USA). A 3-mm silicon (Ecoflex 00-20, Smooth-on, USA) layer was poured around the head to create a surface texture mimicking human skin as previously described [6]. Finally, silicon tubes were inserted through the nose and mouth cavities to create an airtight airway to be connected to a mechanical lung simulator. A more detailed example of the design and a final head model is shown in the Supplemental material, Additional file 1: Figure S2 and S3.

### Mask design

Personalized, oronasal masks were designed as a composition of three parts: a fully customized cushion, a frame and a frame ring. To assemble the mask, the cushion is placed in the frame with fixation through the frame ring (Fig. 1B and Additional file 1: Figure S4). The frame and accompanying frame ring were designed in eight different sizes for children up to 7 years old (see Additional file 1: Figure S5 and 6), as based on the DINED database [19], and on studies of Goto et al. [20], and Young [21]. The inner part of the frame was designed to minimize unwanted (dead space) volume by following the facial landmarks and minimize frame height. For the personalized NIV cushion, we developed a plugin for Rhinoceros (Robert McNeel & Associates, USA, available on request). In this software, the facial 3D scan can be uploaded, and eight facial landmarks are then selected manually (see Supplemental material, Additional file 1: Figure S7). Based on the input, the Rhinoceros software produces a mask curve and automatically selects a 3D cushion model according to the nasion–pogonion distance. For this exploratory study, two different mask sizes (small and large) were chosen for each test head model: the size automatically chosen by the software and one size smaller, to examine the accuracy of the sizing system. The files, which were saved as an STL format, could be sliced and exported to a 3D printer.

The masks are stabilized and fixated to the patient’s head using a 3D printed holder (PC-ISO, Fortus 450mc, Stratasys, Israel) and headgear with five fixation points (Respireo SOFT nasal masks from Air Liquide Healthcare, France), as described previously [6].

### Mask production

The frames were 3D printed in VeroClear material (Objet30, Stratasys, Israel). The frame rings were 3D printed in PC-ISO material (Fortus 450mc, Stratasys, Israel). The personalized cushions were 3D printed in three different biocompatible (a minimum of ISO 10993-5 and 10993-10) soft materials:

1. Silicone urethane (SU) (Sil30, A-35, Carbon 3D, USA) on a DLS printer (M3 Max, Carbon3D, USA), resulting in two masks: SU_small_ and SU_large_,
2. Silicone (Amsil 20501, A-50, Elkem Silicones, Norway) on a FDM printer (3D4Makers, Netherlands and Purpose AM Systems, Latvia), resulting in two masks: Si_small_ and Si_large_
3. Soft photopolymer resin (MED414, A-50, Loctite, Henkel, Germany) on a DLP printer (OriginOne, Stratasys, Israel), resulting in two masks: SPR_small_ and SPR_large_.

**Fig. 2:**
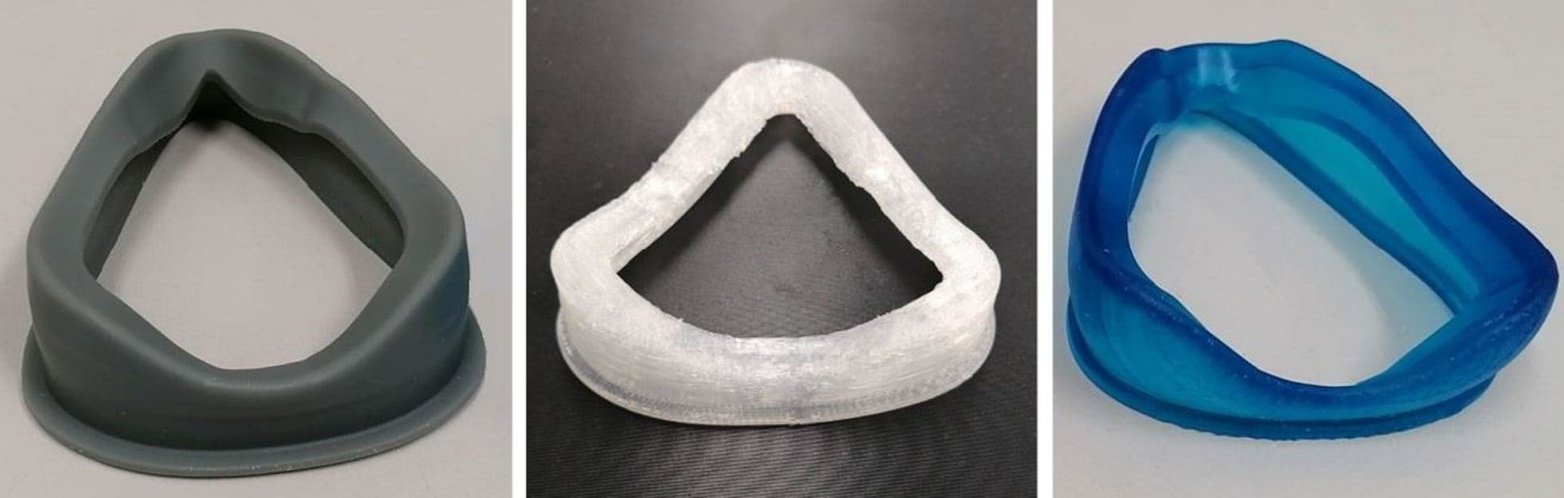
An overview of the personalized cushion materials. From left to right: silicone urethane (SU) printed on a DLS printer, silicone (Si) printed on an FDM printer, and soft photopolymer resin (SPR) printed on a DLP printer

Figure 2 shows examples of masks produced in these different soft materials.

### NIV simulation

Pediatric NIV was simulated in a bench test to determine the performance of the produced personalized masks. As a comparison for the personalized masks, the only commercial oronasal NIV mask available in our PICU (Nivairo, Fisher & Paykel Healthcare, New Zealand, size: XS, which is the smallest available) was also tested. The setup consisted of a head model connected to a mechanical lung simulator (Michigan Instruments, Grand Rapids, USA) (Fig. 1). The test lung simulator was set at 25 ml/cmH_2_O compliance and 20 cmH_2_O parabolic resistance. These settings were fixed during all NIV simulation test runs. The mask was placed and fixated to the head model, and connected to a ventilator (Hamilton C6 ventilator, Hamilton Medical, Switzerland) by a standard dual limb breathing circuit (Fisher&Paykel Healthcare, New Zealand). A standard computer was used to continuously collect the flow (31 Hz), volume (31 Hz), airway pressure (31 Hz) and air leak (2 Hz) data from the ventilator using the Hamilton DataLogger software and to collect the facial surface pressure (2 Hz) data using custom-made software in Python.

For ventilation, the respiratory rate was set on 20 breaths per minute, with an inspiration–expiration ratio of 1:3 in NIV-ST modus, which delivers time-cycled, pressure-supported breaths. The ventilator parameters and facial surface pressures were then measured for 90 s, which thus included analysis of a total of 30 breaths, for three consecutive ventilator pressure steps, noted as peak-inspiratory pressure/positive end-expiratory pressure: 15/5 cmH_2_O, 20/5 cmH_2_O, 25/10 cmH_2_O. For each of these three ventilator pressure steps, we performed three independent measurements (triplicate). Between these measurements, the mask was removed from the head model, re-fixated and the headbands were re-adjusted.

### Performance outcomes

To examine the performance of the masks, air leak percentage (L_air_, reported by the ventilator as Vleak%, which is automatically derived per breath by one minus the calculated exhaled volume divided by inhaled volume, multiplied by 100%), facial surface pressure (N/cm^2^) on the nose bridge (P_nb_), pressure on the chin (P_c_) and average pressure (P_av_, calculated as the mean from all six pressure sensors) of the masks were compared.

### Statistical analysis

Data on the performance outcomes for each mask were collected three times in independent measurements for 30 breaths, and summarized into mean values per measurement. These data are presented as medians (IQR) for each mask per ventilator pressure step. No sample size calculation regarding the number of head models was performed for this exploratory preclinical bench study. Comparisons between multiple mask types were analyzed using the Friedman test for non-parametric repeated measures data due to the small sample size and absence of normal distribution upon graphical histogram presentation or the Shapiro–Wilk test. If this test yielded a significant *p*-value (< 0.05), Wilcoxon signed-rank tests were executed as post hoc comparison between the masks. IBM SPSS Statistics (version 28) was used for the analysis.

## Results

All three soft materials were successfully 3D printed and could be assembled into NIV oronasal masks with an airtight, biocompatible personalized soft cushion. Total production time of these masks was below 15 h: the SU masks (small and large) took around 11 h (2 h to print, 1 h of manual post-processing and 8 h of curing); Si masks (small and large) took 15 (11 h of printing, 4 h of curing and 15 min of post-processing); SPR masks (small and large) took 10 h (9 h to print and 1 h of post-processing and curing).

**Fig. 3:**
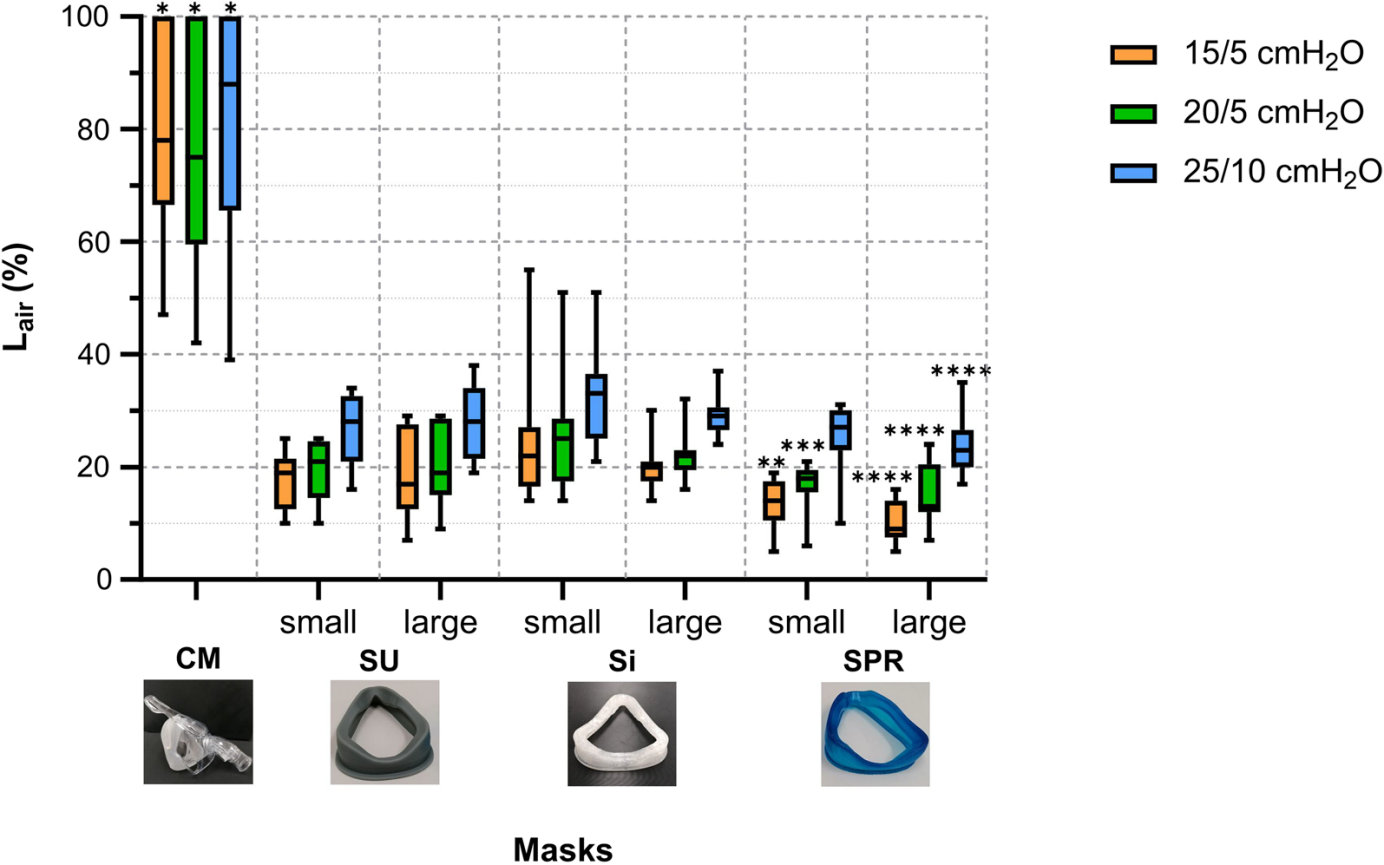
Air leak percentages (L_air_) for the commercial mask (CM) and each personalized non-invasive ventilation (NIV) mask (silicone urethane (SU) small and large; silicone (Si) small and large; and soft photopolymer resin (SPR) small and large) during pediatric NIV bench test simulation at three different ventilation pressure steps (peak-inspiratory pressure/positive end-expiratory pressure: 15/5 cmH_2_O, 20/5 cmH_2_O and 25/5 cmH_2_O). The boxplots and error-bars depict median/IQR and range, respectively. *CM versus SU_small_, SU_large_, Si_small_, Si_large_, SPR_small_ and SPR_large_ (*p* < 0.05); **SPR_small_ versus Si_small_ and Si_large_ (*p* < 0.05); ***SPR_small_ versus Si_small_ (*p* < 0.05); ****SPR_large_ versus Si_small_ and Si_large_ (*p* < 0.05) as analyzed by Friedman non-parametric test with post hoc testing

Air leak performance was generally acceptable for all personalized masks (Fig. 3), and there were no relevant differences between the different head models. Overall, L_air_ was below 30%, except at the 25/10 cmH_2_O ventilation pressure step. In comparison, the commercial oronasal mask (Nivairo) showed leak percentages of > 60% in two test head models (DS and CFCS) even when strapping the headgear to its limits, and > 95% in the VCFS model. In the latter model no sufficient contact of the commercial mask with the face was possible. This caused disruption of the flow and pressure levels in such a way that no reliable NIV could be simulated. At all three ventilator pressure steps (15/5, 20/5 and 25/10 cmH_2_O) all personalized masks had lower L_air_ as compared to the commercial mask (Fig. 3). Moreover, there was a small benefit in the SPR masks as compared to the Si masks. The data on L_air_ per test head model are presented in Additional file 1: Figure S8 and examples of the ventilator waveforms are shown in Additional file 1: Figure S9.

**Fig. 4:**
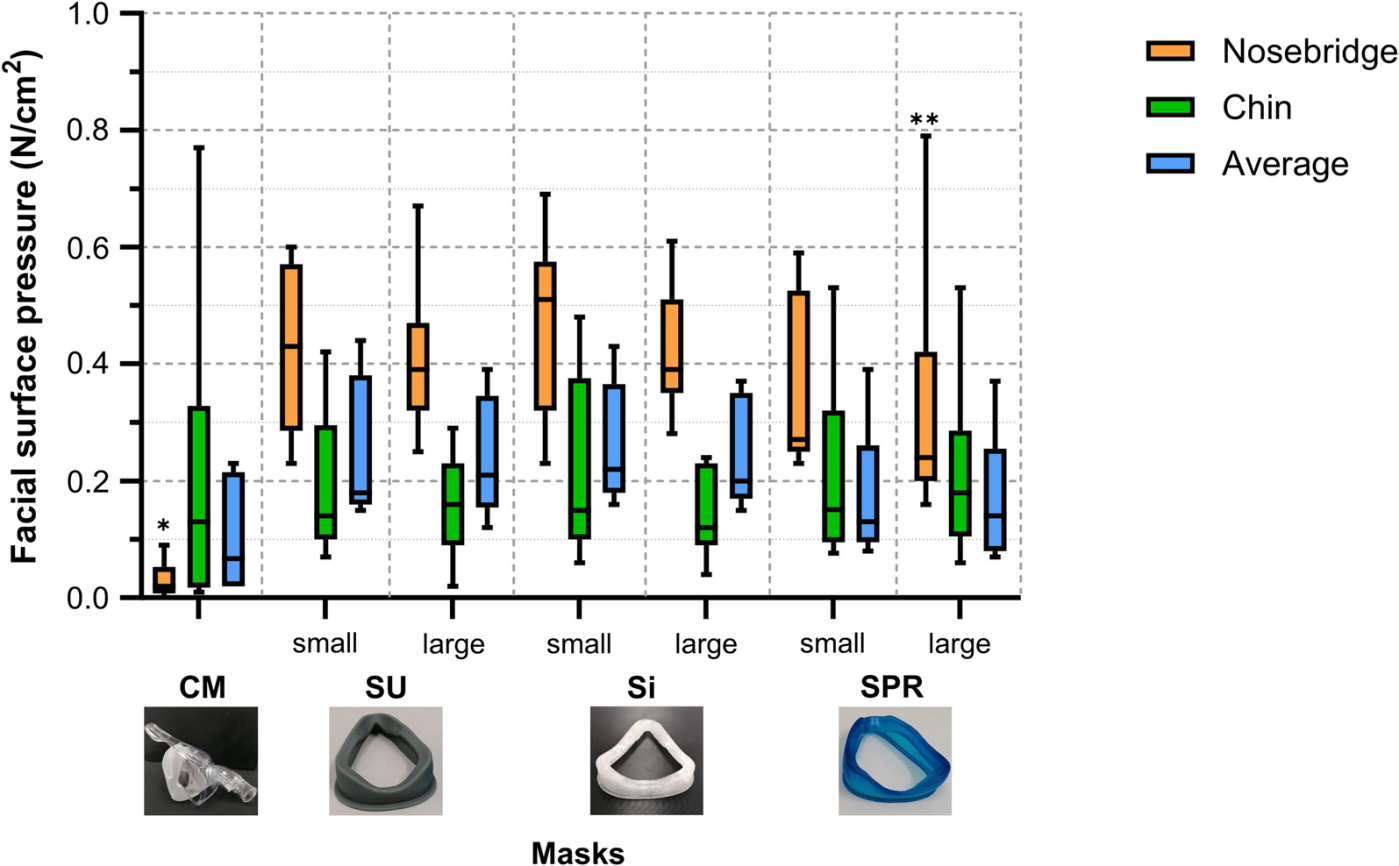
Facial surface pressures (N/cm^2^) (average for all six sensors, nose bridge and chin region) for the commercial mask (CM) and each personalized non-invasive ventilation (NIV) mask (silicone urethane (SU) small and large; silicone (Si) small and large; and soft photopolymer resin (SPR) small and large) during pediatric NIV bench test simulation at a peak-inspiratory pressure of 15 cmH_2_O and a positive end-expiratory pressure of 5 cmH_2_O. The boxplots and error-bars depict median/IQR and range, respectively. *CM versus SU_small_, SU_large_, Si_small_, Si_large_ and SPR_small_ (*p* < 0.05); **SPR_large_ versus SU_small_ and Si_small_ (*p* < 0.05)

Median facial surface pressure values from the personalized masks were below 0.50 N/cm^2^ at 15/5 cmH_2_O ventilator pressure step (Fig. 4). Surface pressure delivered to the nose bridge was relatively high as compared to the chin region. Only P_nb_ showed significant differences between the masks. Herein, the commercial mask resulted in significantly lower facial pressure than SU_small_, SU_large_, Si_small_, Si_large_ and SPR_small_, while values for SPR_large_ were lower than SU_small_ and Si_small_. However, as the commercial mask did not make sufficient contact with the face in the case of the VCFS model, data for this test head were omitted from the analysis. Additional file 1: Figure S10 shows P_nb_, P_chin_ and P_av_ for each ventilator pressure step. The data on facial pressure per test head model are presented in Additional file 1: Figure S11.

## Discussion

The aim of the current study was to test the air leak and facial surface pressure performance of personalized oronasal NIV face masks using different 3D printed soft materials. In this proof-of-concept bench simulation of pediatric NIV using head models of young children with abnormal facial characteristics, we show that it is possible to obtain biocompatible, personalized mask cushions with acceptable air leak and surface pressure performance using a relatively short and semi-automated production process.

To our knowledge this study is the first to examine different 3D printed biocompatible soft materials to develop personalized NIV masks for children, aimed at supporting critically ill children with acute respiratory failure. For application in the acute setting, personalized masks should be rapidly and readily available after initial stabilization with commercial NIV masks or alternative interfaces. As such, ideally the total production time of a personalized mask should be as low as possible, with little hands-on time to decrease the need for available or specialized personnel. For this purpose, the relatively novel technique of 3D printing soft, biocompatible materials holds much promise [22, 23]. It is believed that this method provides a benefit as compared to recent studies that used molding techniques [9, 24], or that used 3D printing of rigid materials [18, 25]. In our current production process, we were able to develop personalized NIV masks within a 12-h time window (the SPR mask). Considering that a facial 3D scan by handheld devices can be made within 60 s, and can be uploaded into our semi-automated software for mask selection, ongoing medical device development appears justified. However, before implementing personalized NIV masks in critically ill children, pilot (first-in-human) and subsequent clinical testing is warranted. It will, in particular, be important to identify pediatric patient subgroups (e.g., young age) that will most likely benefit the greatest of personalized masks in terms of NIV success. In this light, it is important to note that the syndromes associated with facial abnormalities included in our study were chosen to facilitate proof-of-concept, but do not necessarily represent populations receiving more specific attention.

In terms of air leak, all personalized NIV masks, performed to an acceptable degree (leak percentage generally below 30%) [26] in our bench test using head models with specific facial features. The observed air leak was significantly lower in the personalized interfaces versus the commercial mask and among the personalized mask there appeared to be a small advantage of the masks with photopolymer resin material (the SPR mask). In case of the SPR_large_ mask, the median (IQR) L_air_ was as low as 9 (8–14)%. Although moderate air leak is usually compensated for by ventilators, in particular with pressure targeted ventilation [10], large unintentional leak can result in decreased patient–ventilator synchronization, discomfort (e.g., by air flow to the eyes) and reduced alveolar ventilation, subsequently contributing to treatment failure [11, 13, 26, 27]. To reduce air leak from an ill-fitted mask, a common maneuver by nurses in daily practice is to tighten the mask headgear straps. This has the downside to increase pressure applied to the skin, contributing to development of painful sores [8]. On the other side, too low leak percentages due to a personalized fit may carry the increased risk for rebreathing of CO_2_ [10, 28].

Impaired facial skin integrity during NIV in children, resulting in painful sores and ulcers, is a common complication due to high mask pressure [7]. Overall, in our model the personalized oronasal masks resulted in average surface pressures below the capillary pressure of 0.44 N/cm^2^ (33 mmHg) in our head models, which is deemed necessary to prevent pressure injury [29]. Here, also the SPR masks slightly performed better in comparison to the other soft materials. This average pressure was comparable to that exerted by the very thin, soft silicone texture of the commercial mask. Nevertheless, in the personalized masks, the pressure at the nose bridge in some measurements exceeded this threshold. Previous studies on facial pressures of NIV masks in adults reported pressures on the nose bridge of 0.3–1.4 N/cm^2^ [30]. Compared to these values, a median of 0.24 N/cm^2^ (IQR 0.22–0.42) of the SPR_large_ mask appears fairly low. Since the nose bridge is most prone to pressure injury [4], future personalized mask designs, including headgear modifications, should continue to focus on lowering P_nb_. However, it should be taken into account that there is a fine balance between air leak and skin pressure: when accepting higher leak percentages, the pressures applied to the skin could drop. Total face masks, which cover the eyes, nose and mouth, may distribute skin pressure more evenly over a larger surface excluding the nose bridge. However, in children these masks often have large air leaks at the top side, and they have the disadvantage of risk of claustrophobia and eye irritation [31]. Nasal masks, which also avoid the nose bridge region, have the clear disadvantage of increased air leak when opening the mouth, and in critically ill children with acute respiratory failure this type of mask is usually insufficient to provide high positive bi-level pressures.

This study has several limitations. First, there are several inherent difficulties with bench testing using NIV simulation [32]. While our head models were designed to evaluate air leak percentage and potential pressure applied to skin, no breathing effort or triggering, which could affect performance of the masks, was simulated. Also, the inner head design was not fully matched to normal upper respiratory tract anatomy, and we did not incorporate different mechanical properties of the simulated respiratory system in our testing. Second, movements of patients during NIV, causing shifting of masks could for obvious reasons not be simulated. For this reason, we performed multiple independent measurements with re-adjustment of the masks in between. Third, in our head models we applied a skin-like layer from silicon that resembles the elasticity of actual skin. While skin thickness varies across the face, this setup used an evenly distributed layer of 3 mm. This could lead to higher estimated pressures in softer facial areas, such as the cheeks, but also in lower estimated pressures at the chin and nose bridge. Fourth, the test setup did not include a nasogastric tube, commonly used in these patients, which may affect mask fit causing additional air leaks [33].

## Conclusion

In this proof-of-concept bench simulation of pediatric NIV using head models with abnormal facial features, we report the development of 3D printed biocompatible, personalized oronasal masks that perform with acceptable air leak and facial surface pressure. Further safety and pilot research in children, along with refinement of the logistical production process, will be necessary to inform future studies that focus on the clinical (cost-)effectiveness of using personalized NIV masks in the treatment of critically ill children.

### Supplementary Information

**Additional file 1.** Supplementary figures.

## Data Availability

The datasets used and/or analyzed during the current study, as well as our developed plugin for mask design are freely available upon reasonable request.

## Abbreviations

PICU: Pediatric intensive care unit
NIV: Non-invasive ventilation
DS: Down syndrome
CFCS: Cardiofaciocutaneous syndrome
VCFS: Velocardiofacial syndrome
SU: Silicone urethane
Si: Silicone
SPR: Soft photopolymer resin
L_air_: Air leakage (%)
P_nb_: Pressure on the nose bridge (N/cm^2^)
P_chin_: Pressure on the chin (N/cm^2^)
P_av_: Average pressure (N/cm^2^)
