# Supplementary material for: Development of personalized non-invasive ventilation masks for critically ill children: a bench study
Source: Intensive Care Med Exp. 2024 Mar 1;12:21. doi: 10.1186/s40635-024-00607-w (PMC10904697; doi:10.1186/s40635-024-00607-w)

**Supplemental content to:**

Development of personalized non-invasive ventilation masks for critically ill children: a bench study, by Pigmans et al.

Index

| **Content** | **Pg.** |
| --- | --- |
| **eFigure 1.** Test head models | 2 |
| **eFigure 2.** Digital design of the components of a pediatric head model | 3 |
| **eFigure 3.** Example of the end result of a pediatric head model | 4 |
| **eFigure 4** Digital design of the components of a personalized ventilation mask | 5 |
| **eFigure 5.** Overview of anthropometric database and sizing system | 6 |
| **eFigure 6.** Mask frame sizes and dimensions | 7 |
| **eFigure 7.** Overview of the software plugin in Rhinoceros. | 8 |
| **eFigure 8.** Air leak percentages of the ventilation masks per test head model | 9 |
| **eFigure 9.** Examples of the ventilator waveforms of the ventilation masks | 11 |
| **eFigure 10.** Facial surface pressures delivered by the different masks at various ventilation pressures | 13 |
| **eFigure 11.** Facial surface pressures delivered by the different masks at various ventilation pressures per test head model | 15 |

**eFigure 1.** Test head models


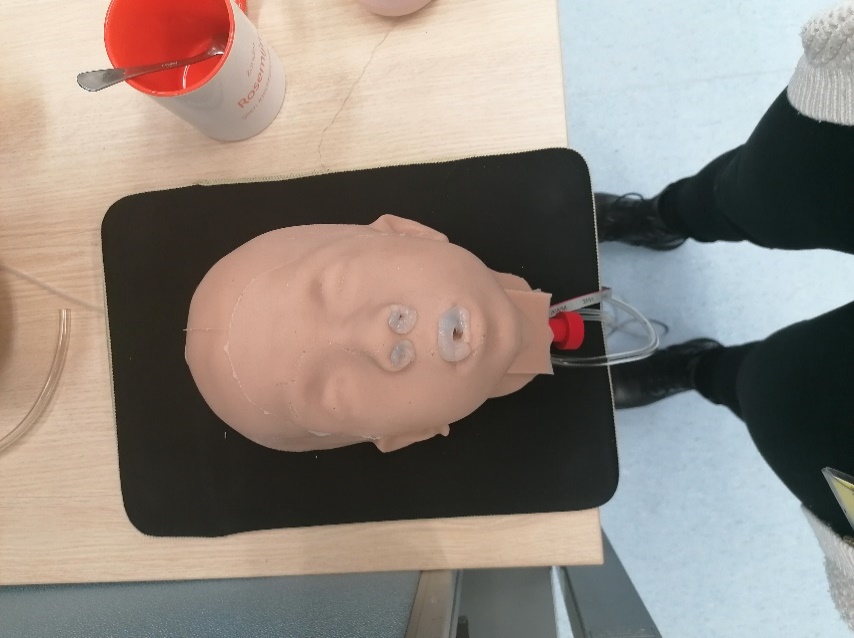

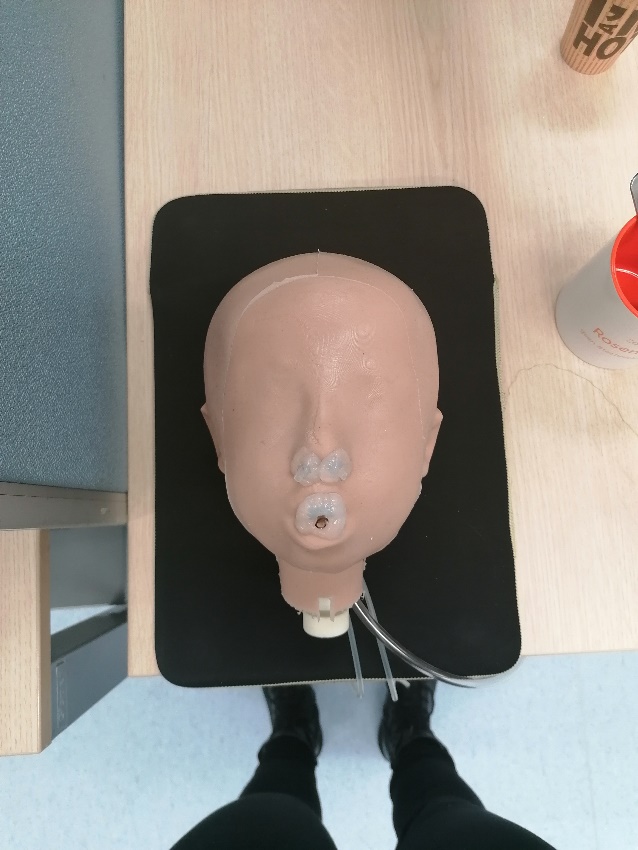

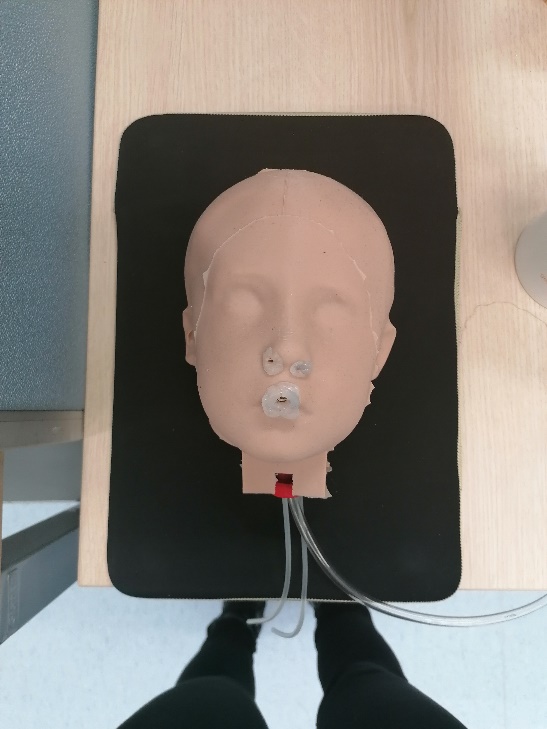

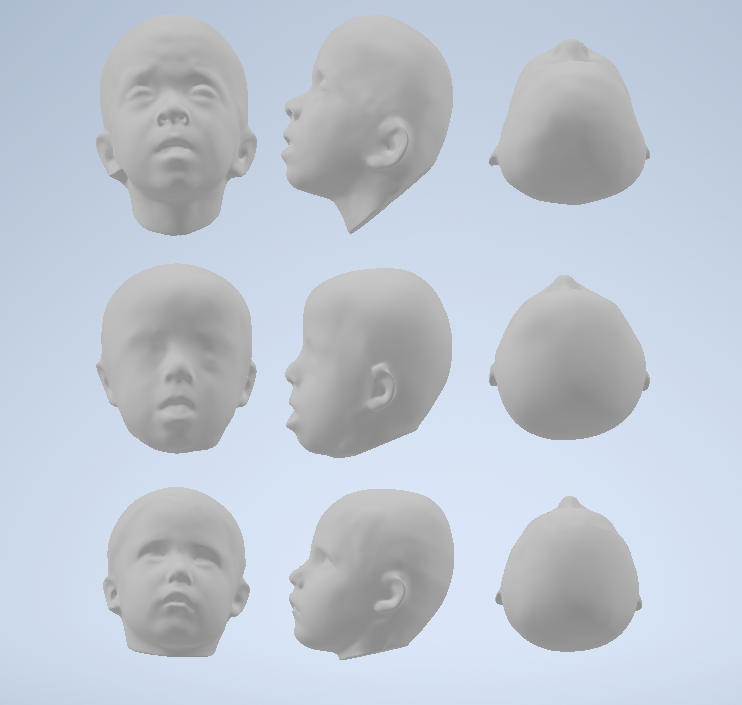

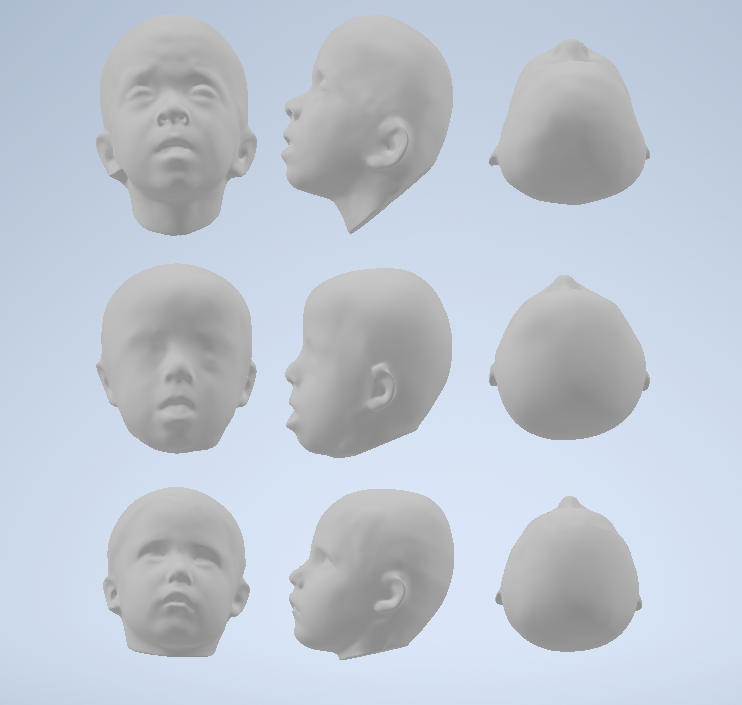

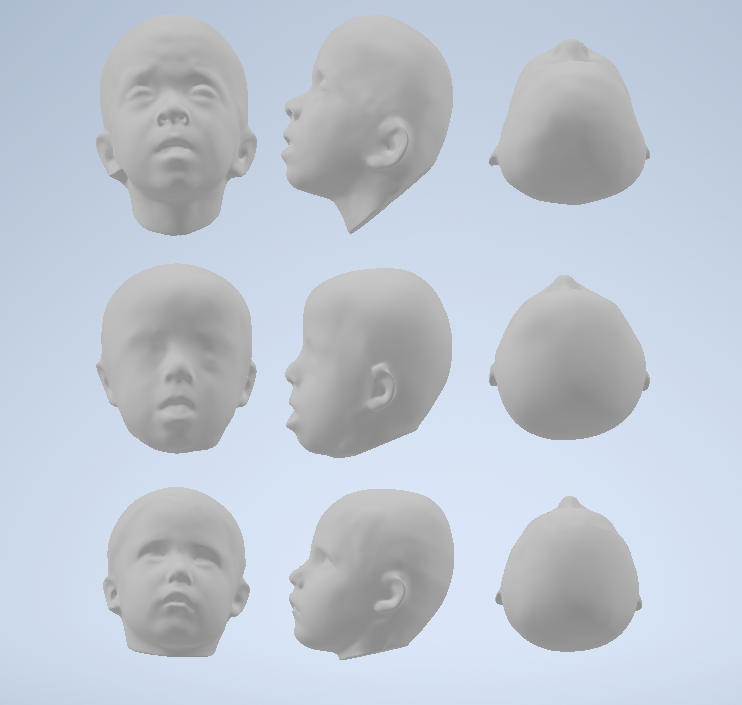


The three test head models of children with different syndromes presented at different angles. From top to bottom: cardiofaciocutaneous syndrome, down syndrome and velocardiofacial syndrome.

**eFigure 2.** Digital design of the components of a pediatric head model


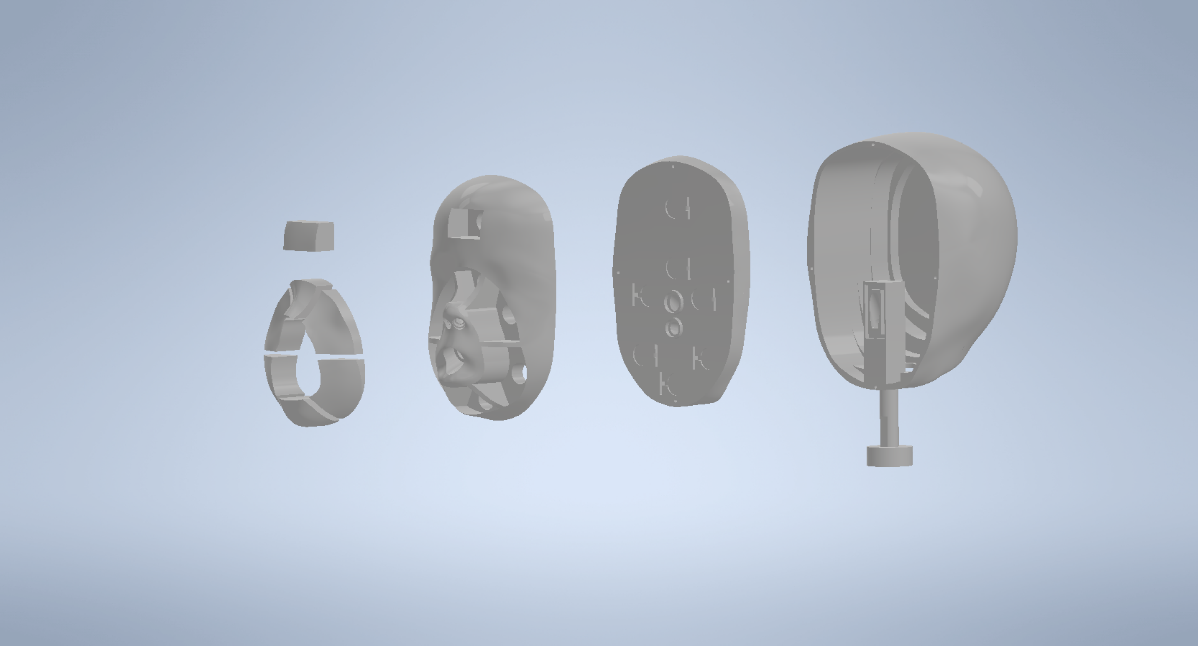


**2**

**1**

**3**

**4**

The design of a head model, composed of four parts: the facial pressure components (1), the face (2), the middle part (3) and the back of the head (4)

**B**

**eFigure 3.** Example of the end result of a pediatric head model
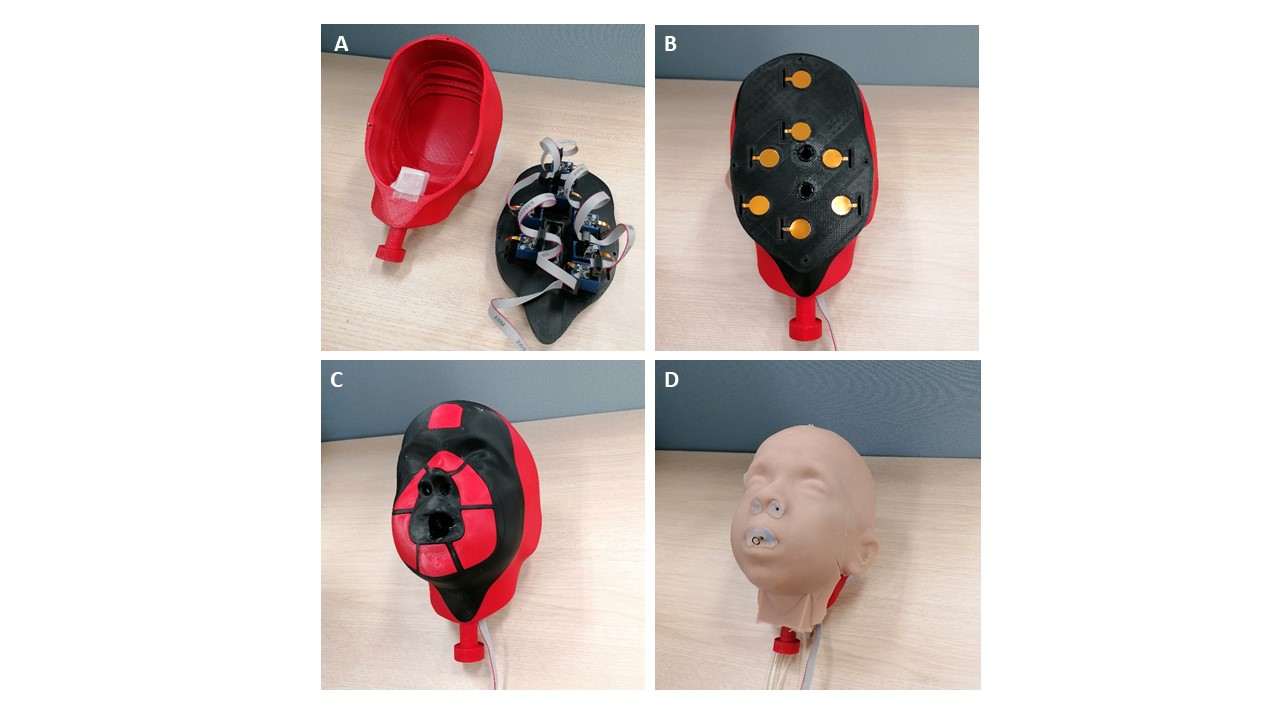


A. The electronics of the resistive pressure sensors (4.5N, calibrated, Singletact, PSS UK Limited, UK) are hidden in the back of the head; B. The sensors are placed on the outline of the masks; C. Total model with facial components for the pressure sensors in place; D. An evenly distributed, 3 mm silicon (Ecoflex 00-20, Smooth-on, USA) layer around the head model to mimic skin textures and silicon tubes inserted to create an airtight setup.

**eFigure 4.** Digital design of the components of a personalized ventilation mask


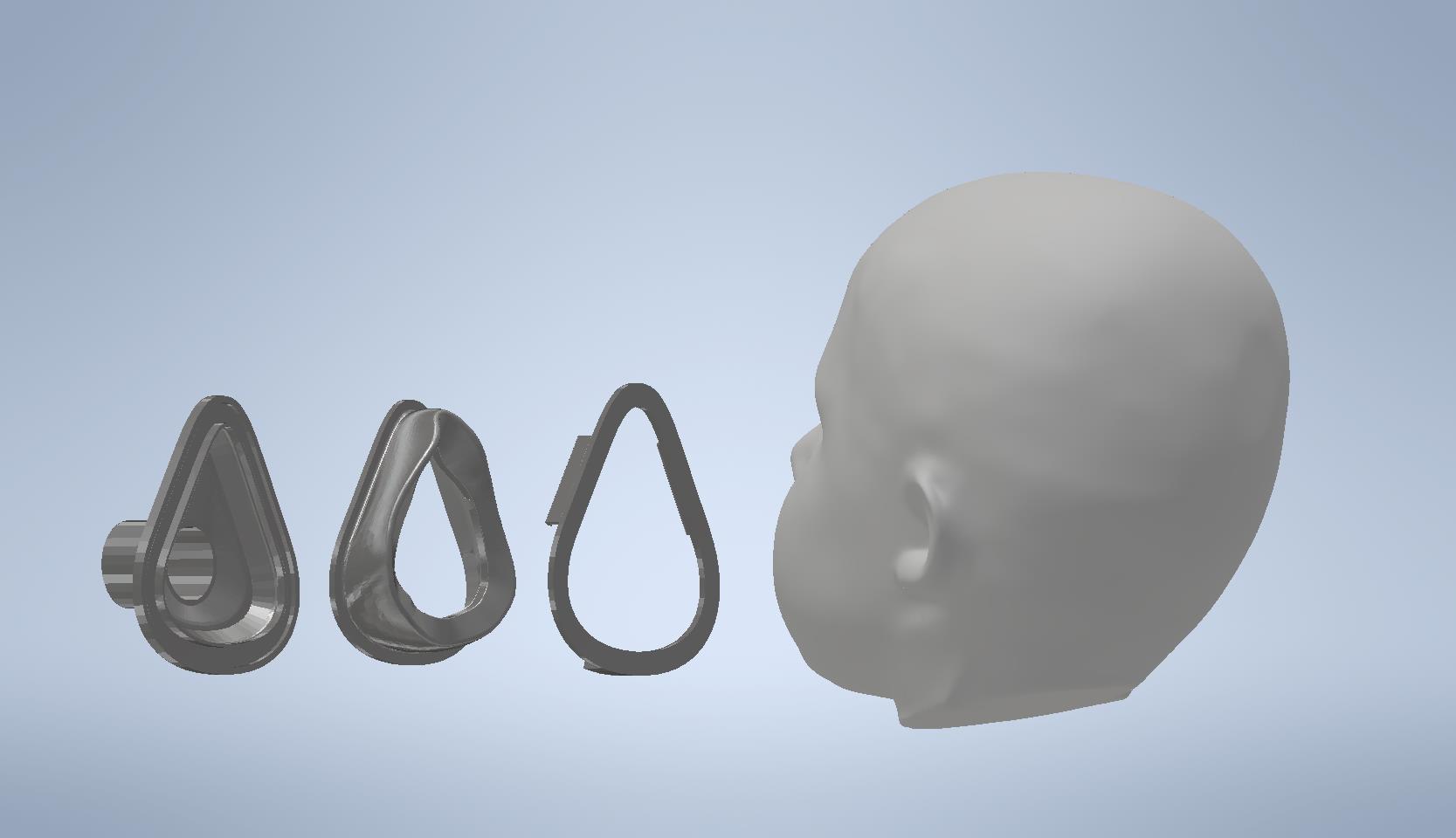


**1**

**2**

**3**

The design of a personalized mask, composed of three parts: 1. the frame, which can be connected to a standard ventilator breathing circuit, 2. the personalized cushion, modelled in our developed plugin (nSize, Netherlands) for Rhinoceros (Robert McNeel & Associates, USA) and 3. the frame ring, which attaches the cushion to the frame.

**eFigure 5.** Overview of anthropometric database and sizing system


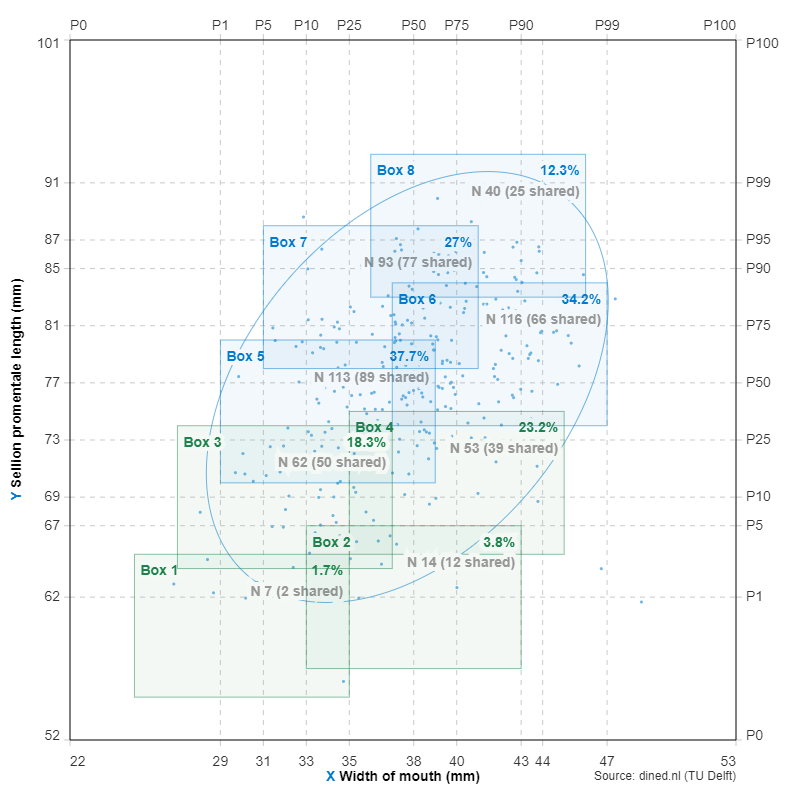


An overview of the eight anthropometric sizing boxes, based on the sellion promentale length (y-axis) and mouth width (x-axis) in mm, on which the mask frame and frame ring dimensions were based. The green and blue boxes are targeted respectively for children <10 kg and >10 kg. Box 3 to 8 are based on the 95% confidence interval of the data collected by Goto (2019) [1] available in the DINED database [2] of Dutch children in the age range of 1-7 years old. The youngest children in this database were 6 months old limiting the accuracy of the sizes for infants. Therefore, Box 1 and 2 were added in DINED, based on the outliers of the database and the measurements of 1 month old children by Young (1966) [3].

1. Goto L, Lee W, Molenbroek JFM, Cabo AJ, Goossens RHM. (2019) Traditional and 3D scan extracted measurements of the heads and faces of Dutch children. International Journal of Industrial Ergonomics 73. doi:10.1016/j.ergon.2019.102828.

2. DINED anthropometric database (2023) TU Delft, Delft <https://dined.io.tudelft.nl/en/ellipse/tool> Accessed 25 Sep 2023.

3. Young JW. (1966) Selected facial measurements of children for oxygen-mask design. AM Rep Apr:1-11.

**eFigure 6.** Mask frame sizes and dimensions


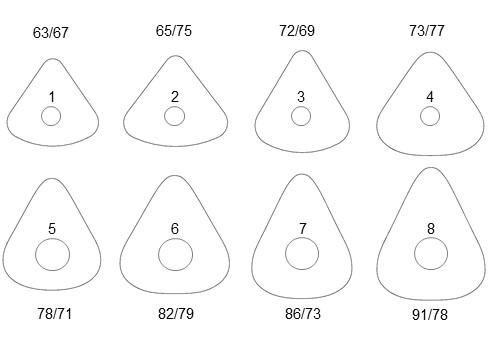


An overview of the dimensions of the mask frames. The top row is size 1-4, designed for the group of children <10kg that require a smaller ventilator tubing system. The bottom row is size 5-8 designed for children >10kg. Each size corresponds with the box of the same number shown in eFigure 3 of this supplement. The height and width of the outer contours of each mask frame is noted in mm (h/w). The height of the frame was calculated as the lower border of the corresponding box plus 8 mm for framing, and the width is the right border of the box plus 20 mm for the flanges of the cushion and plus 12 mm for framing.

**eFigure 7.** Overview of the software plugin in Rhinoceros.
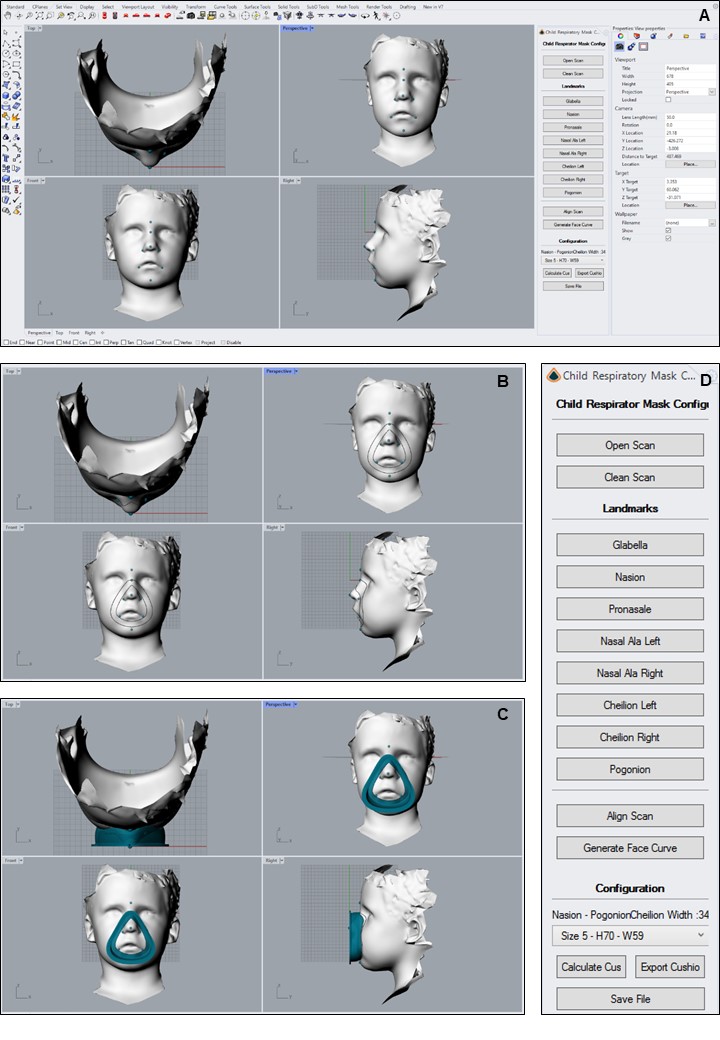


Example of the semi-automated Rhinoceros software with: A. Selection of the eight landmarks within a facial 3D scan; B. Alignment of the scan and generation of the face curve; C. Calculation of the cushion and projection on the face. The size is chosen automatically according to the nasion-pogonion-chelion width when selecting the “Calculate cushion” button; D. Detail of the control panel of the software system.

**
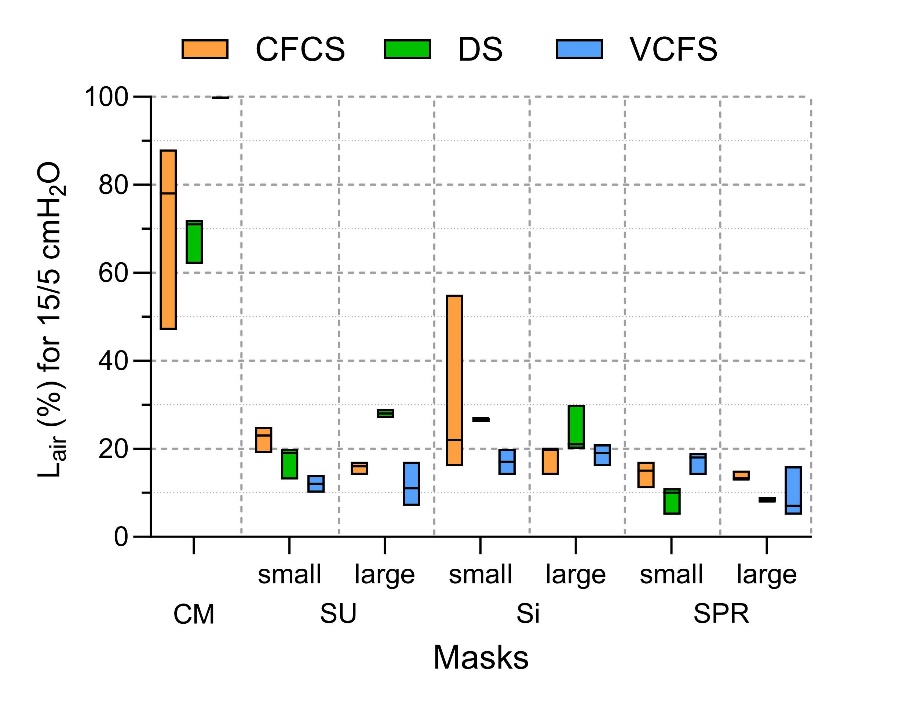
eFigure 8.** Air leak percentages of the ventilation masks per test head model


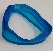


SPR


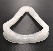


Si


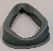


SU


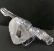


CM

**
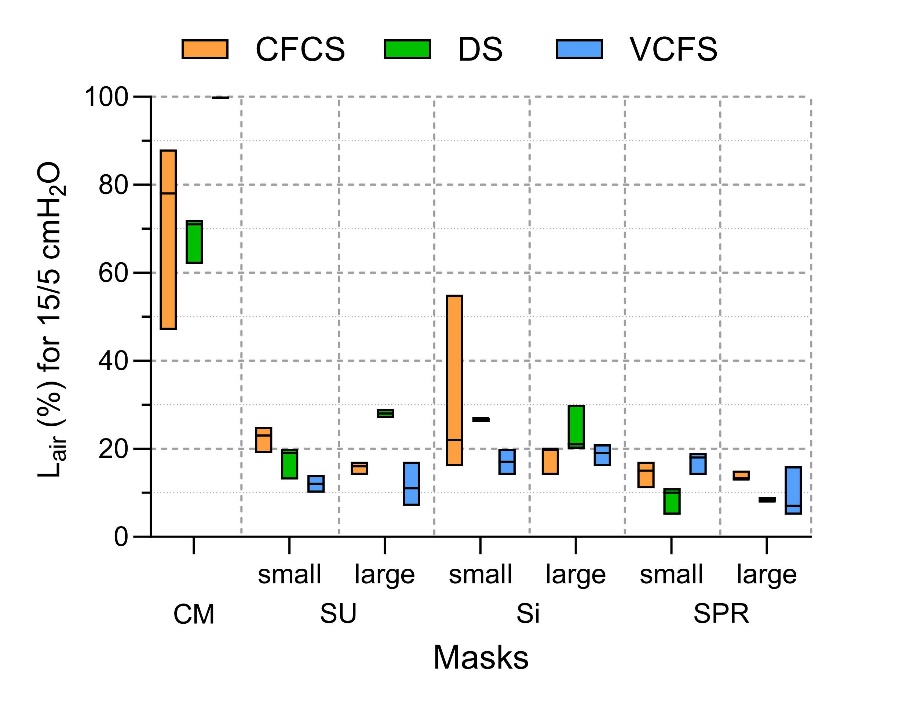
**

**
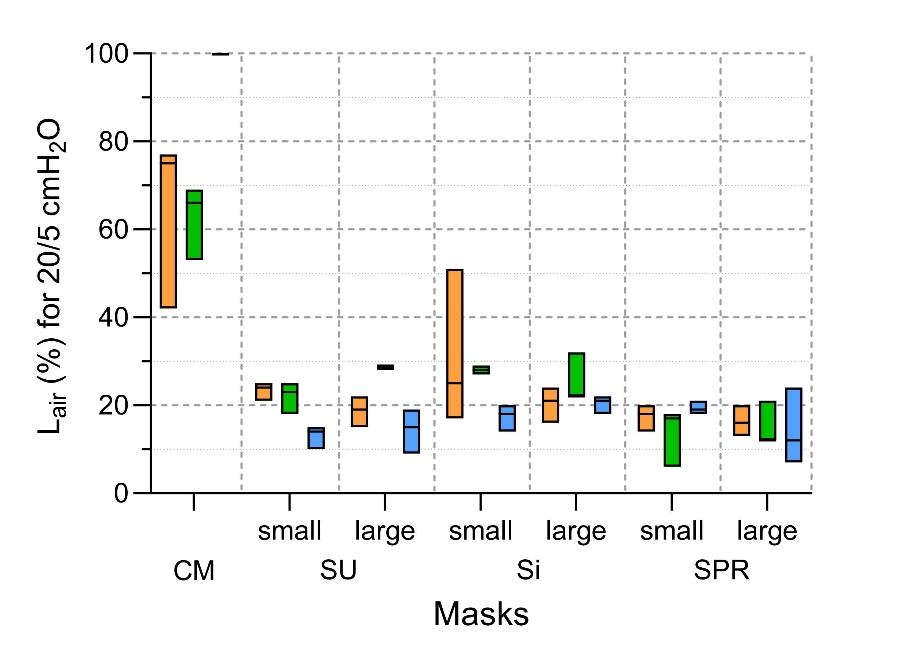
**

**
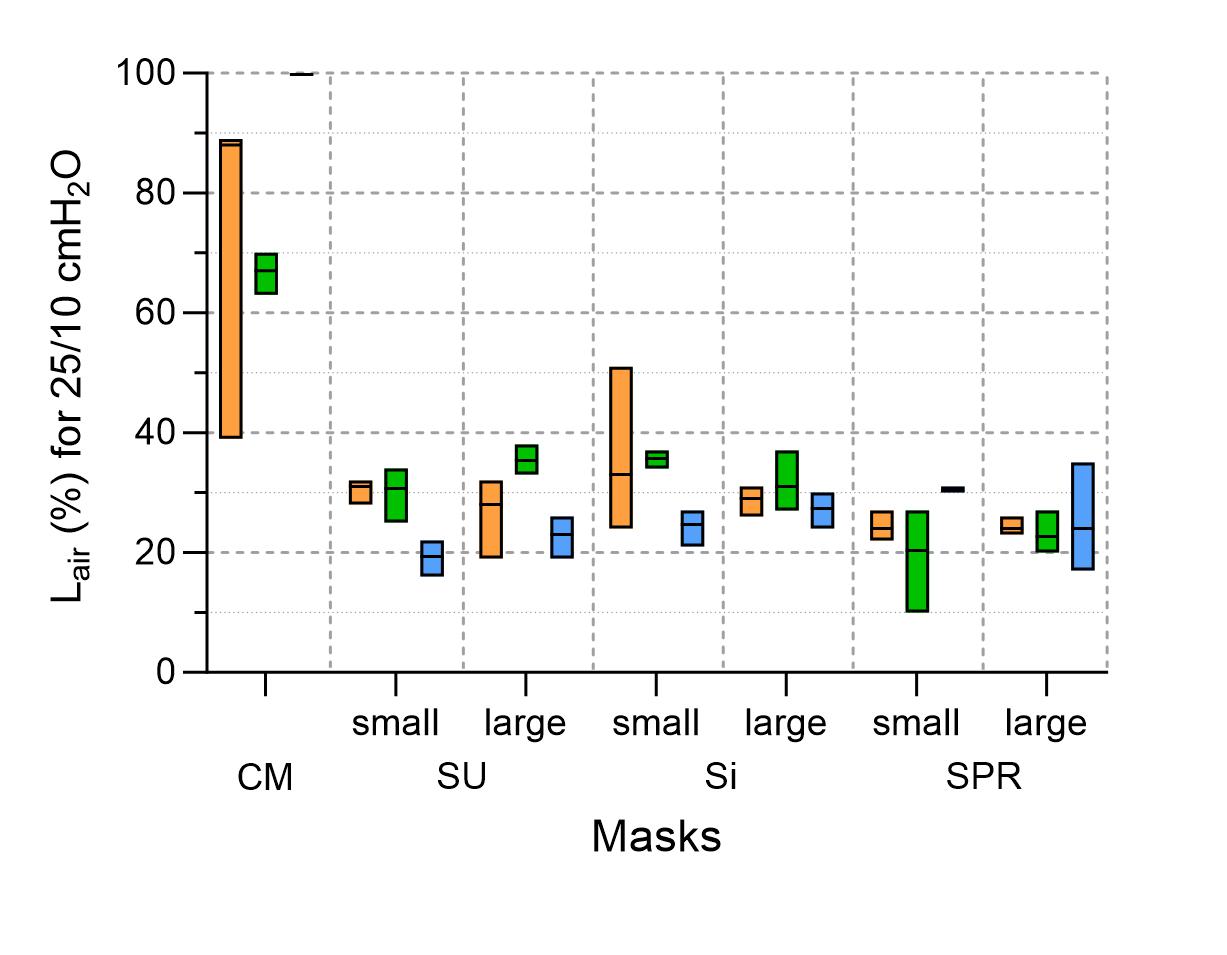

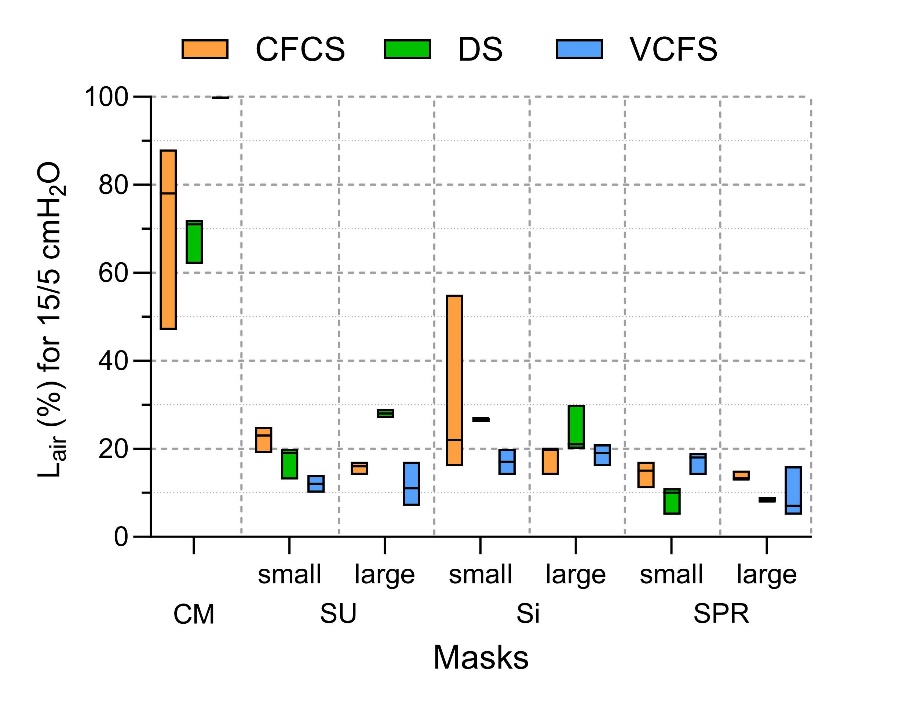
**


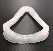


Si


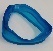


SPR


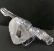


CM


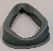


SU

Air leak percentages (L_air_) for the commercial mask (CM) and each personalized non-invasive ventilation (NIV) mask (Silicone Urethane (SU) small and large; Silicone (Si) small and large; and Soft Photopolymer Resin (SPR) small and large) during pediatric NIV bench test simulation in three different test head models (cardiofaciocutaneous syndrome (CFCS), down syndrome (DS) and velocardiofacial syndrome (VCFS). From top to bottom, these data are presented at three different ventilation pressure steps (Peak-Inspiratory Pressure/Positive End-Expiratory Pressure: 15/5 cmH_2_O, 20/5 cmH_2_O and 25/5 cmH_2_O). The boxplots depict median, min and max respectively. The results suggest that there were no major differences in the results obtained from the three different head models.

**eFigure 9.** Examples of the ventilator waveforms of the ventilation masks


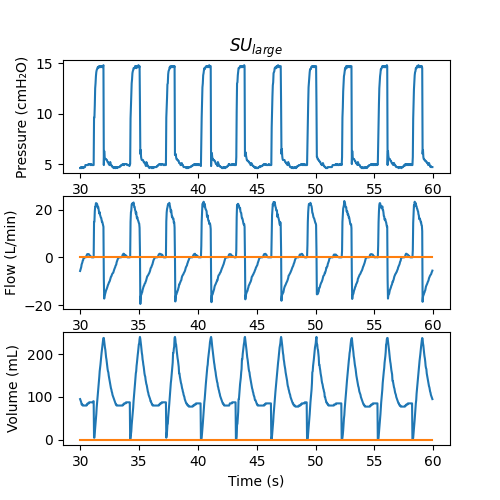


15%


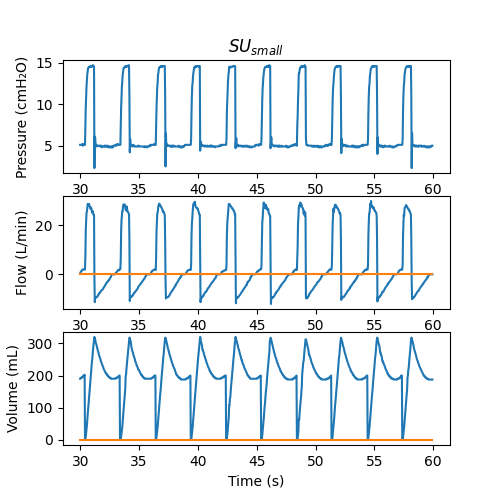


24%


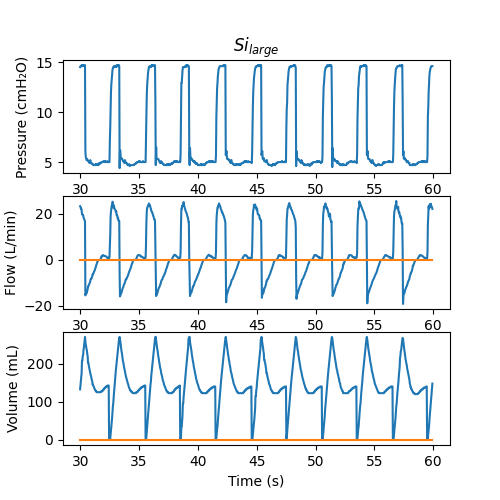


24%


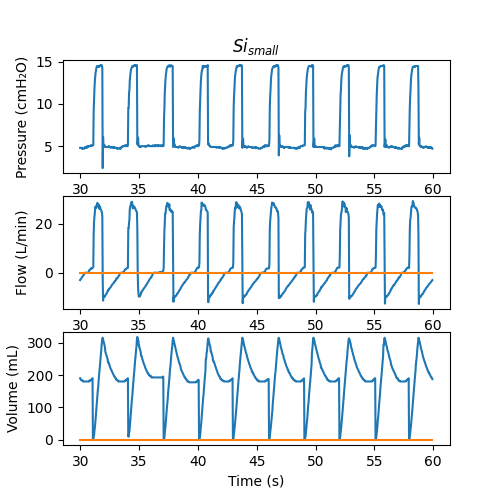


25%


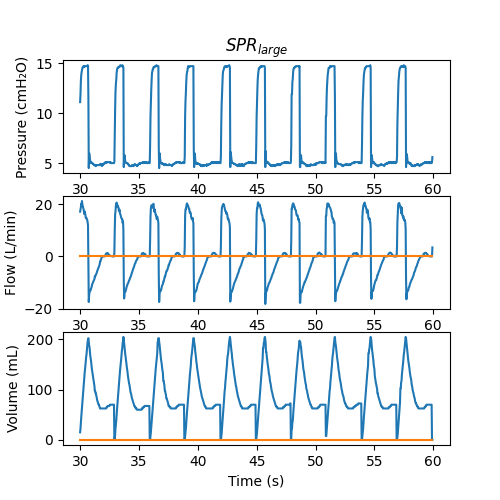


20%


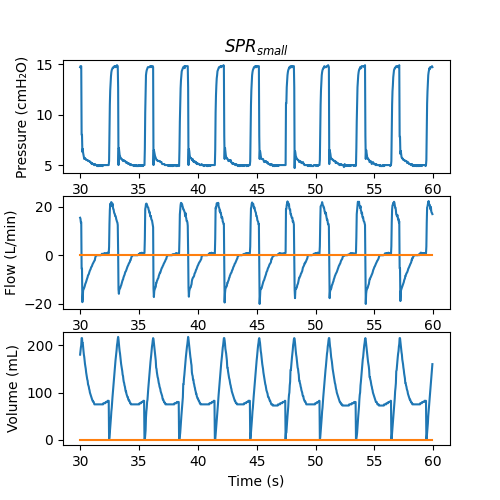


18%

24%

Examples of the ventilator waveforms presented for each ventilator mask (Silicone Urethane (SU) small and large; Silicone (Si) small and large; and Soft Photopolymer Resin (SPR) small and large, and the commercial mask (CM)). All waveforms correspond to the test head model with the cardiofaciocutaneous syndrome at ventilator pressure step 15/5 cmH_2_O. The air leak percentage as produced by the ventilator (Vleak%) is shown in the upper right corner of the volume curve. Vleak% is automatically derived per breath by one minus the calculated exhaled volume (VTe) divided by inhaled volume (VTi), multiplied by 100%. The algorithm of the ventilator calculates VTe based on both the volume and flow curve, which takes into account that the presence of air leak during inhalation also results in air leak during exhalation. The volume curve alone overestimates leak and therefore, the VTe used for Vleak% calculation is an addition of the measured VTe and the estimated exhalation leak volume


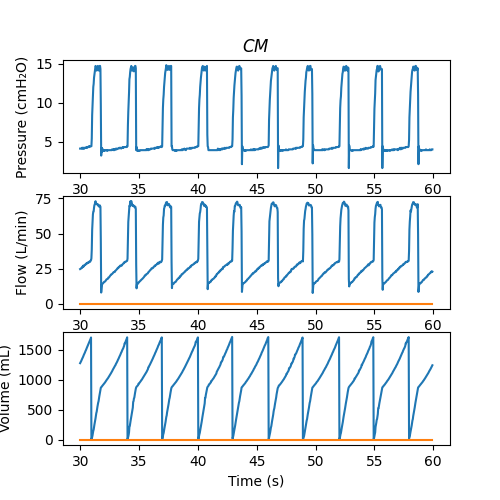


75%

**eFigure 10.** Facial surface pressures delivered by the different masks at various ventilation pressures


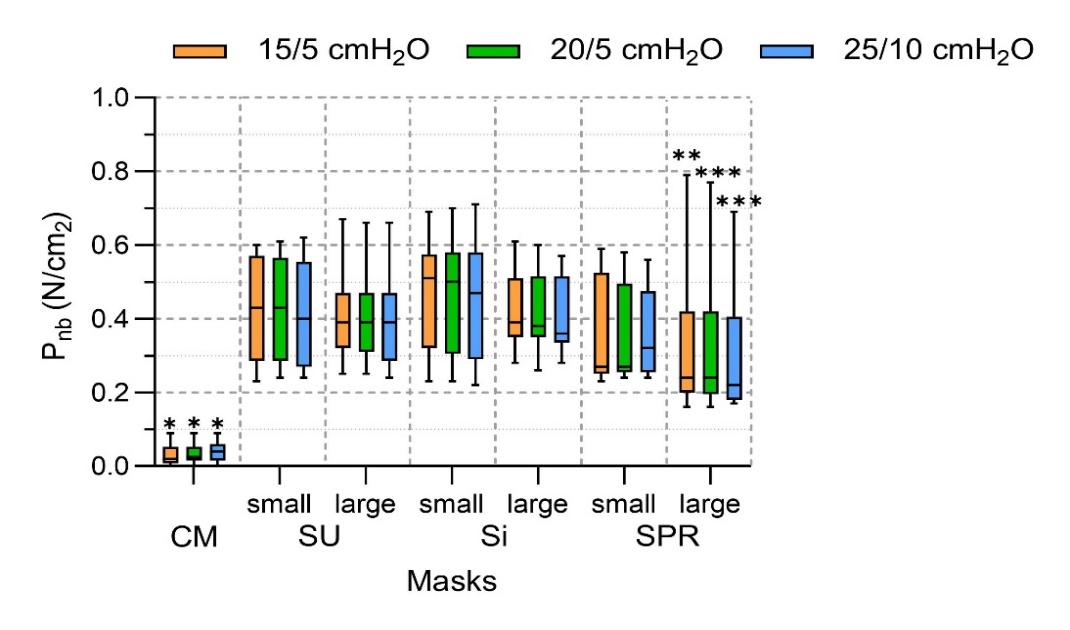

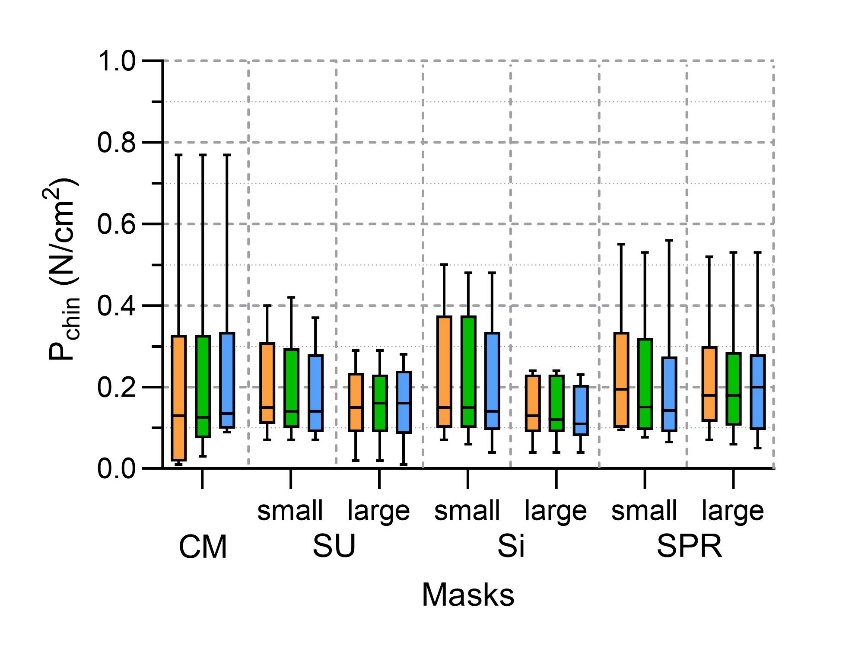

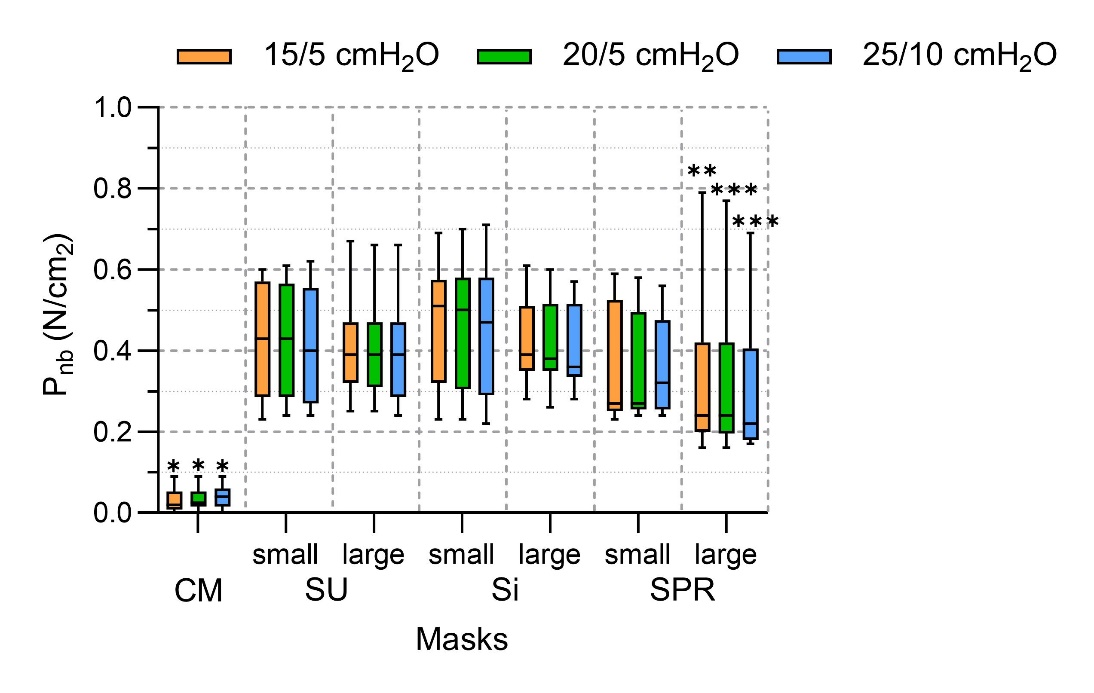

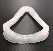


Si


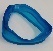


SPR


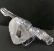


CM


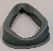


SU


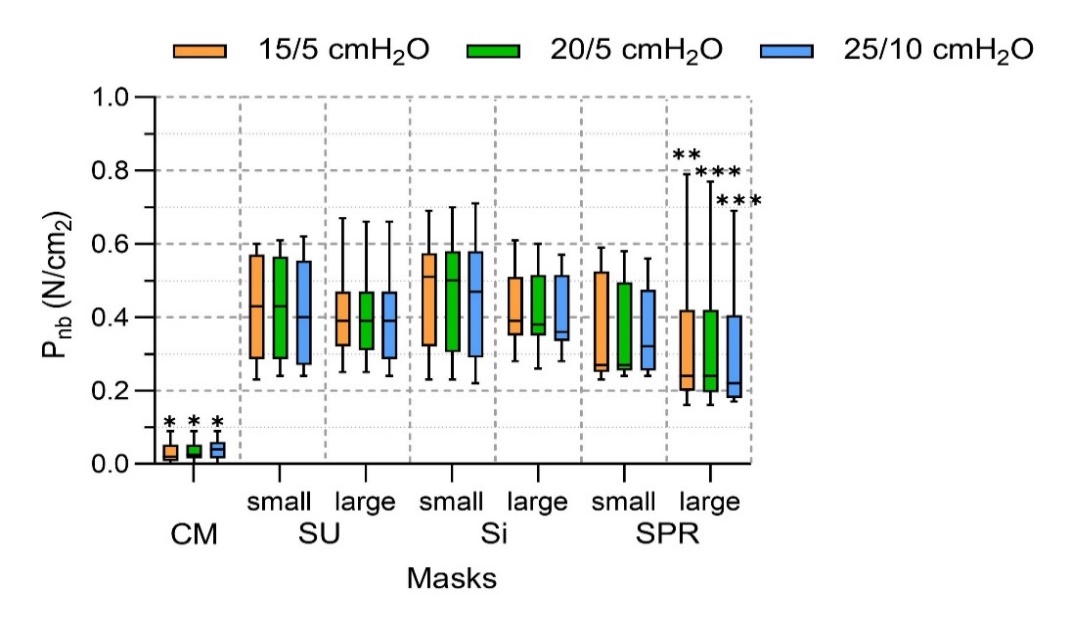


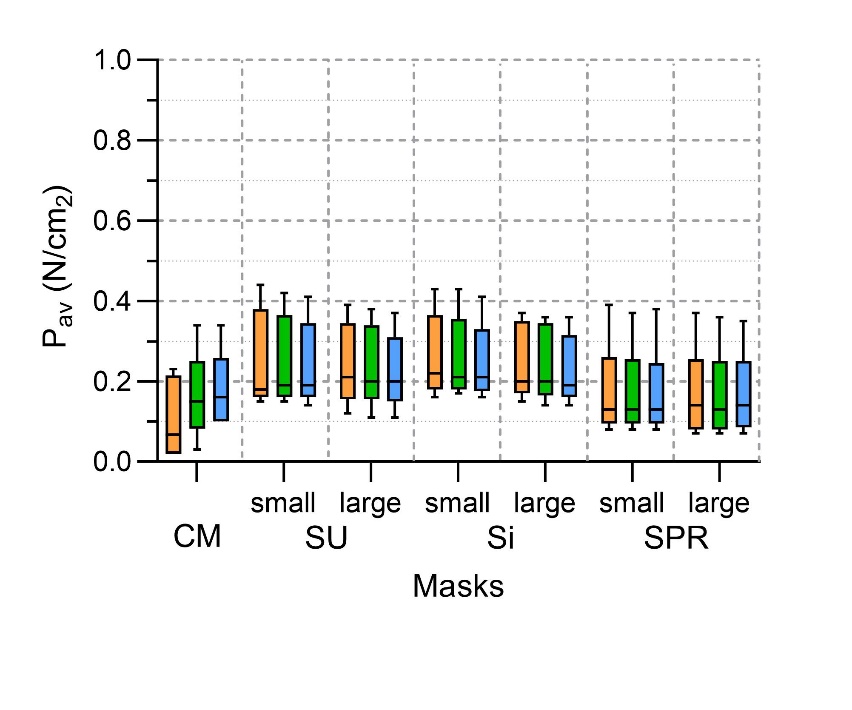


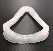


Si


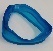


SPR


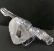


CM


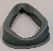


SU

Resulting facial surface pressures (N/cm^2^) (top to bottom: at the nose bridge sensor; at the chin sensor; average of all six sensors) delivered by the commercial mask (CM) and different personalized masks (Silicone Urethane (SU) small and large; Silicone (Si) small and large; and Soft Photopolymer Resin (SPR) small and large) during pediatric non-invasive ventilation bench test simulation at three different ventilation pressure steps (Peak-Inspiratory Pressure/Positive End-Expiratory Pressure: 15/5 cmH_2_O, 20/5 cmH_2_O and 25/5 cmH_2_O). The boxplots and error-bars depict median/IQR and range respectively. Note: the data of CM only included two test head models, as the velocardiofacial syndrome model had no sufficient contact of the commercial mask with the face due to improper fit. *Commercial versus SU_small_, SU_large_, Si_small_, Si_large_ and SPR_small_ (p<0.05); **SPR­_large_ versus SU_small_ and Si_small_ (p<0.05); ***SPR­_large_ versus SU_small_, Si_small_ and Si_large_ (p<0.05) as analyzed by Friedman non-parametric test with post-hoc testing.

**eFigure 11.** Facial surface pressures delivered by the different masks at various ventilation pressures per test head model


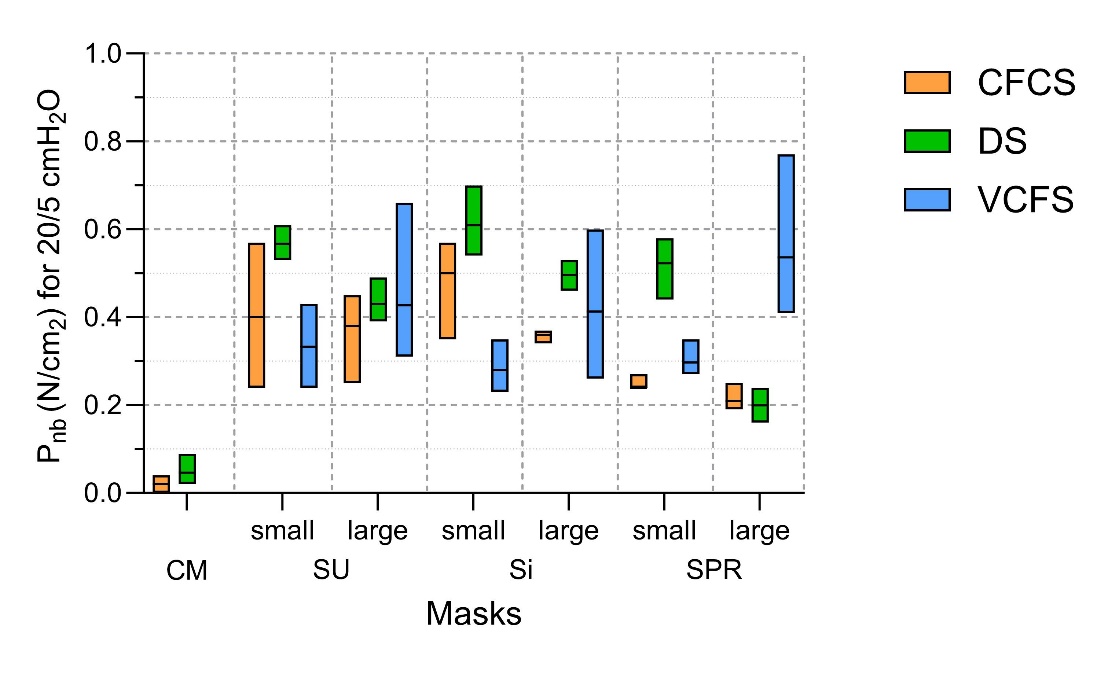

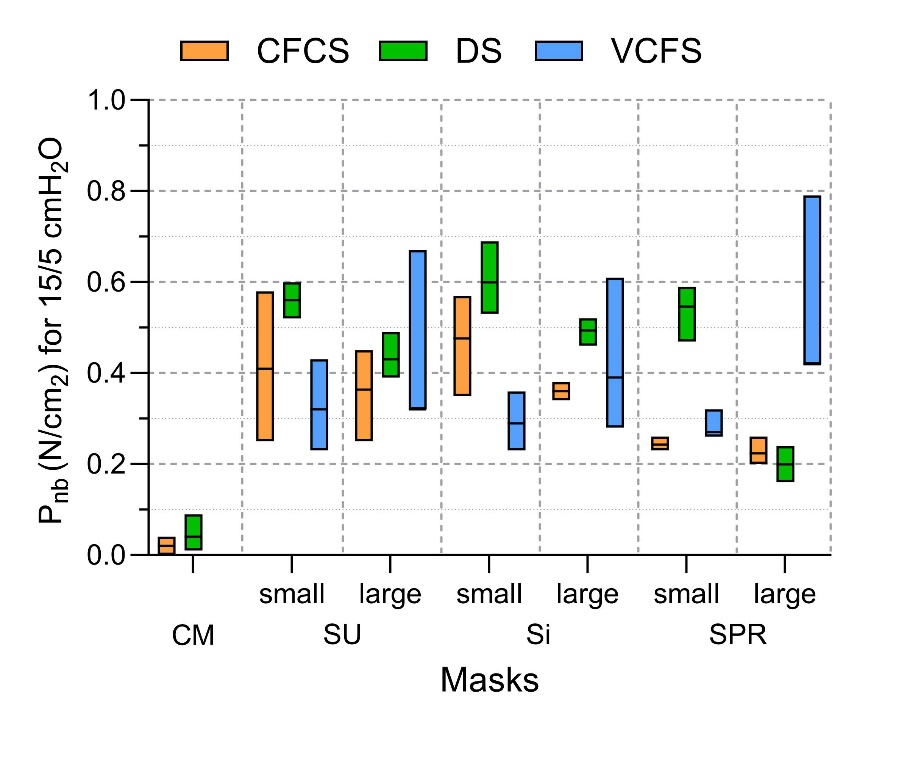


SU


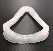


Si


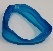


SPR


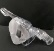


CM


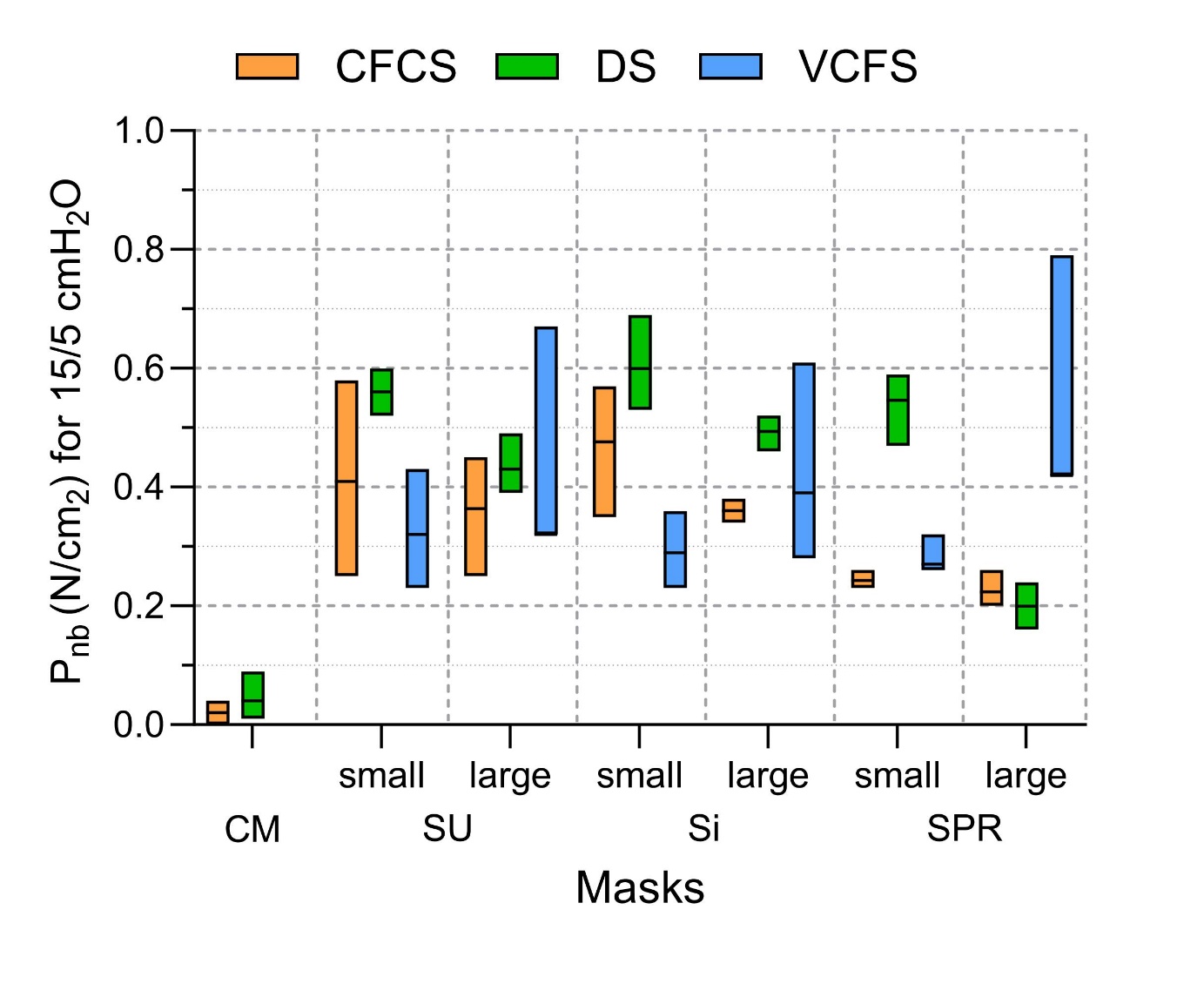

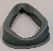

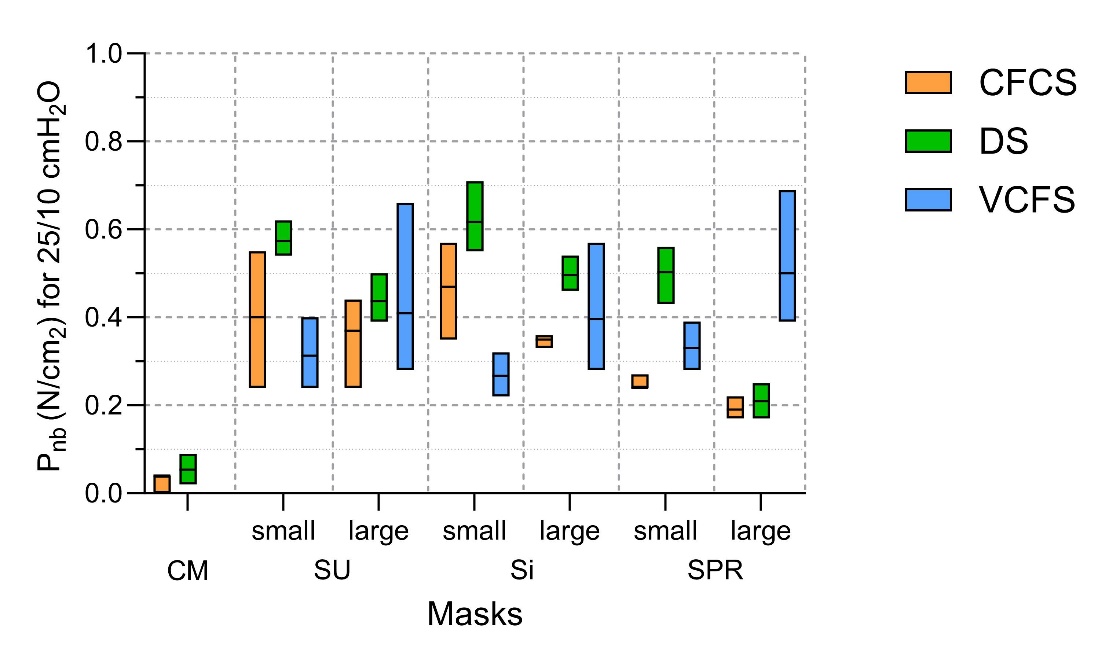

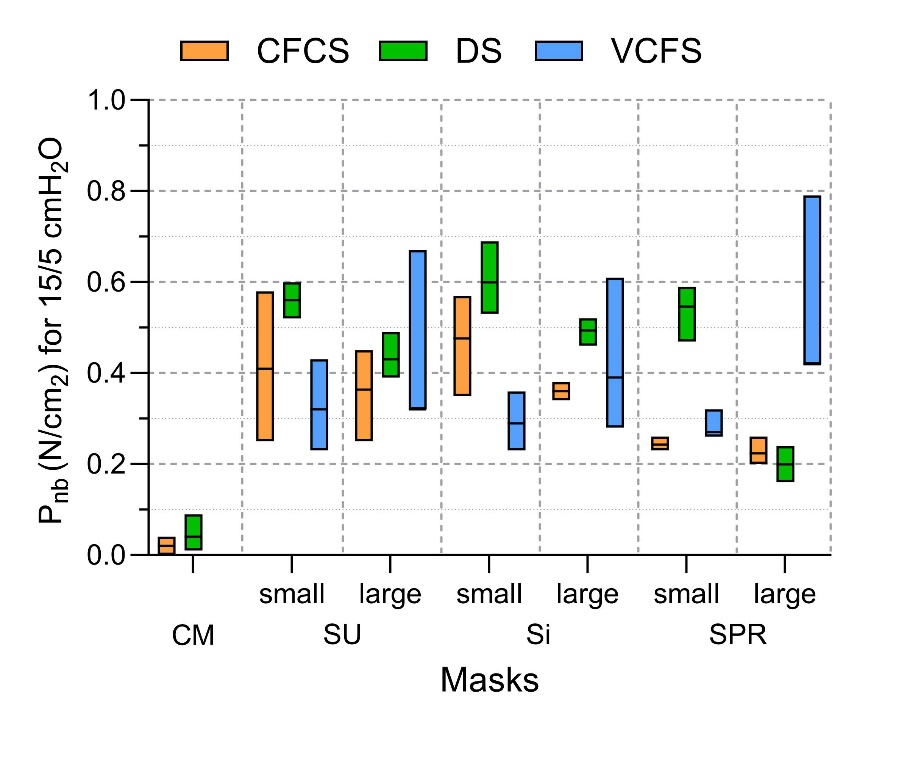


SU


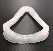


Si


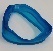


SPR


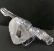


CM


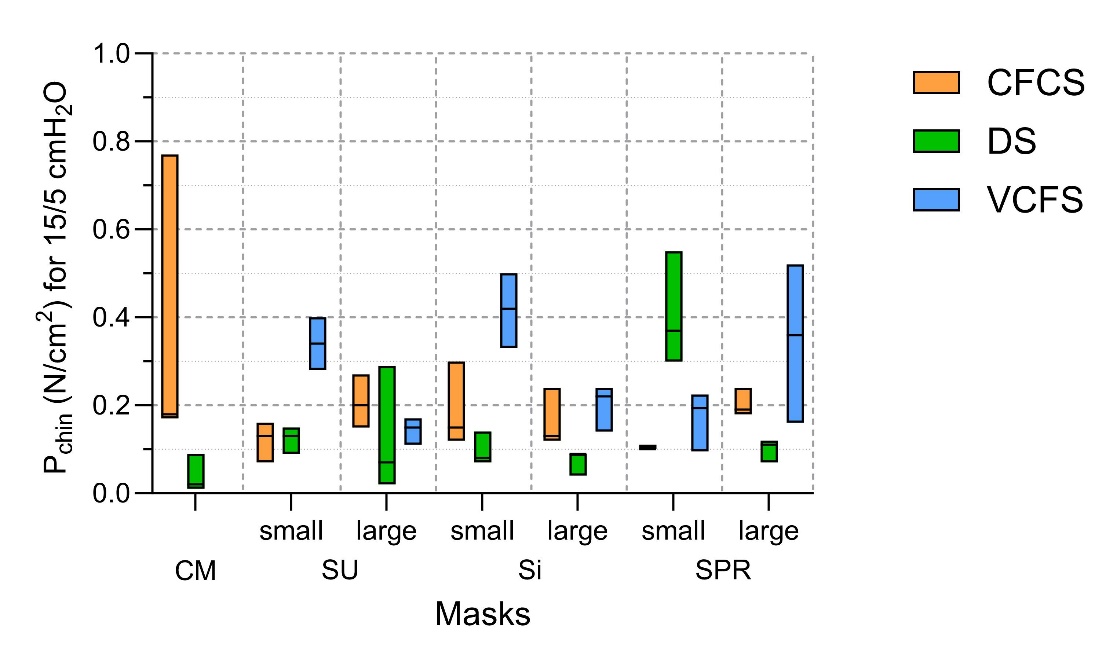

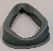

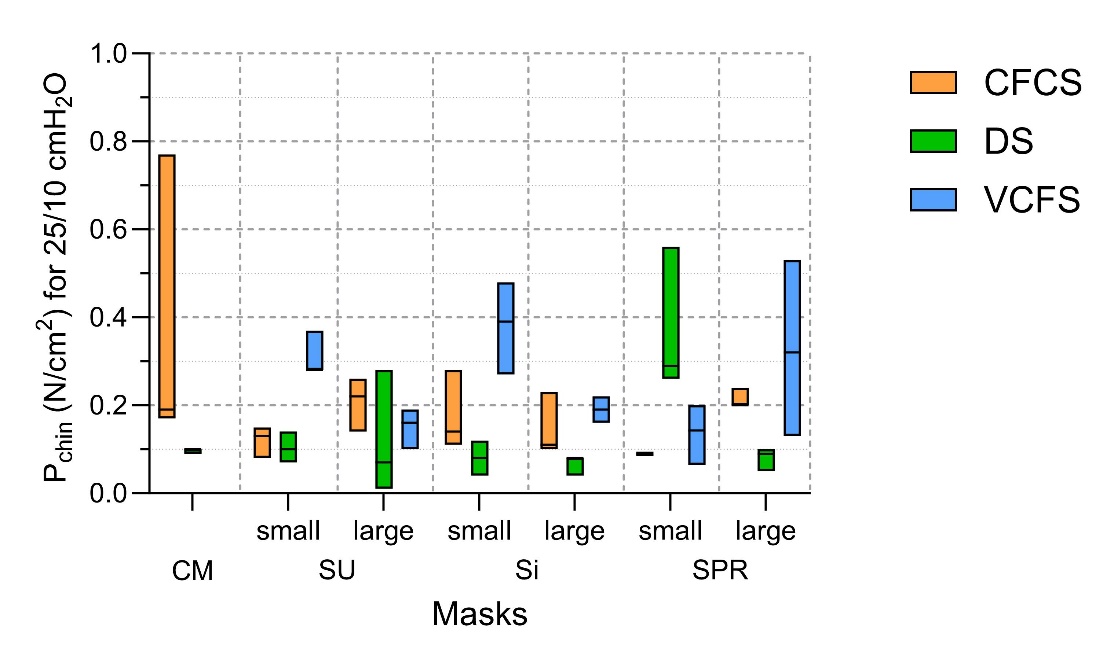

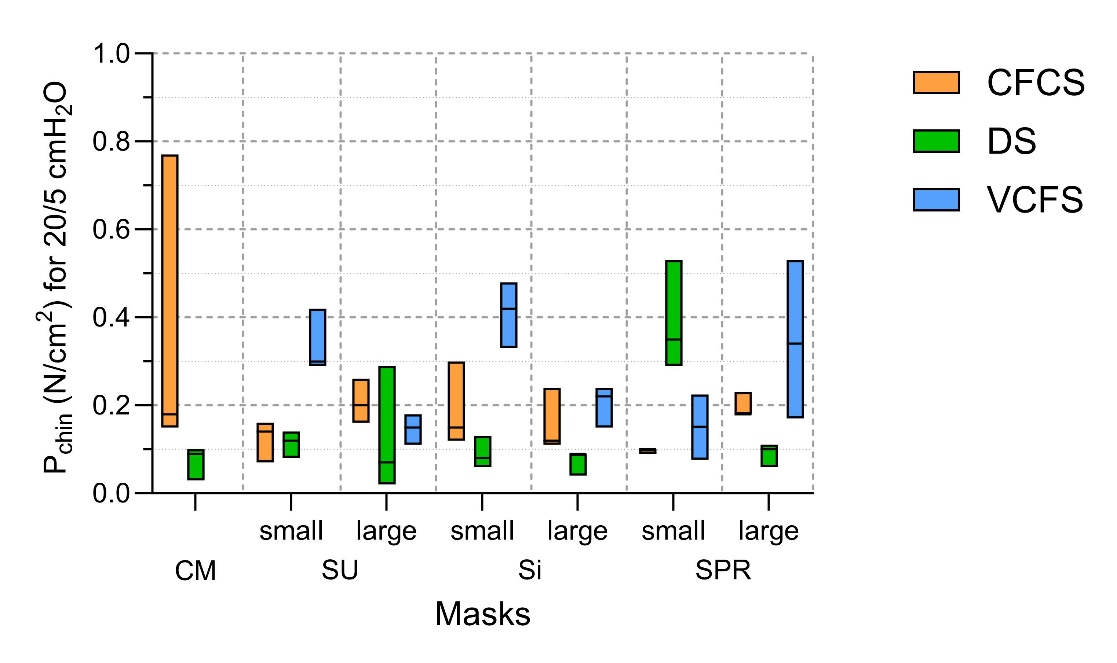

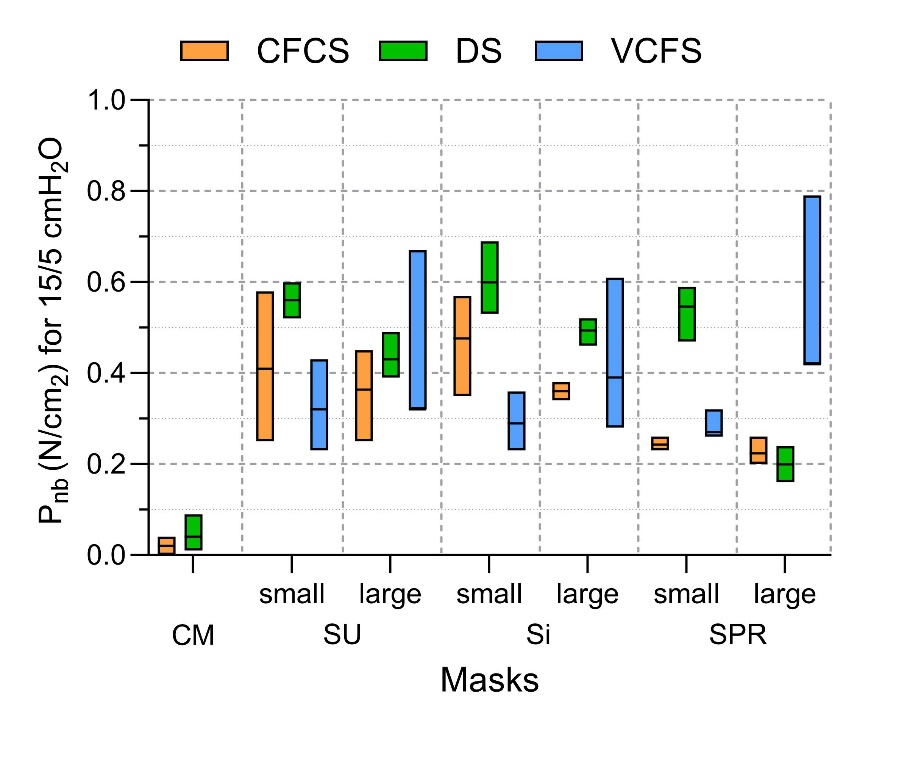


SU


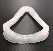


Si


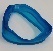


SPR


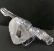


CM


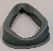

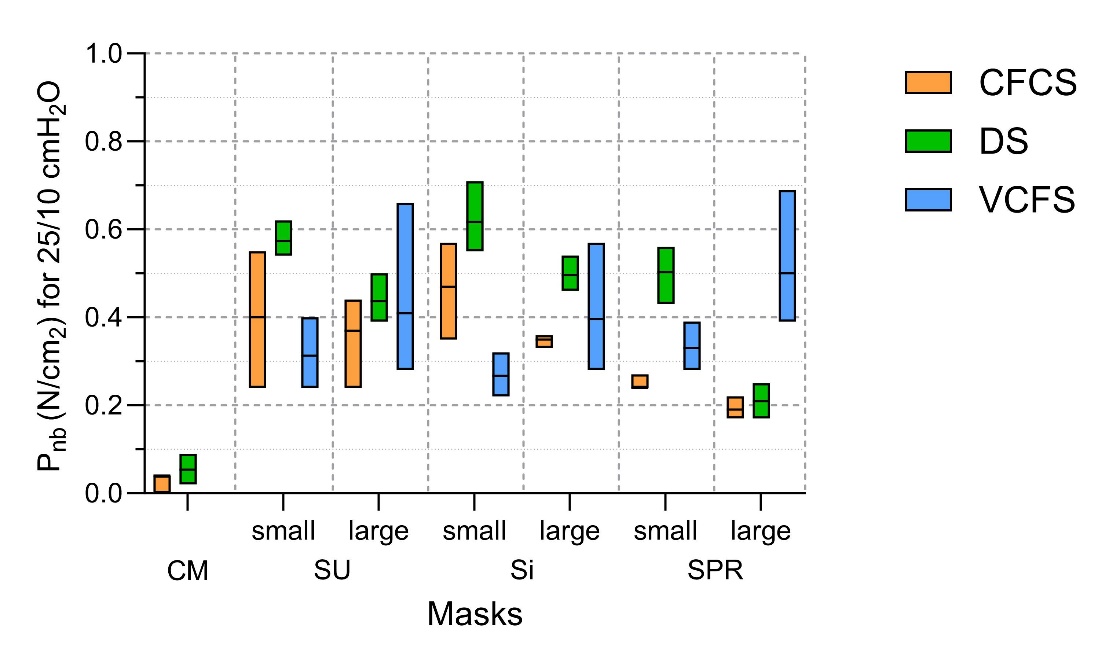

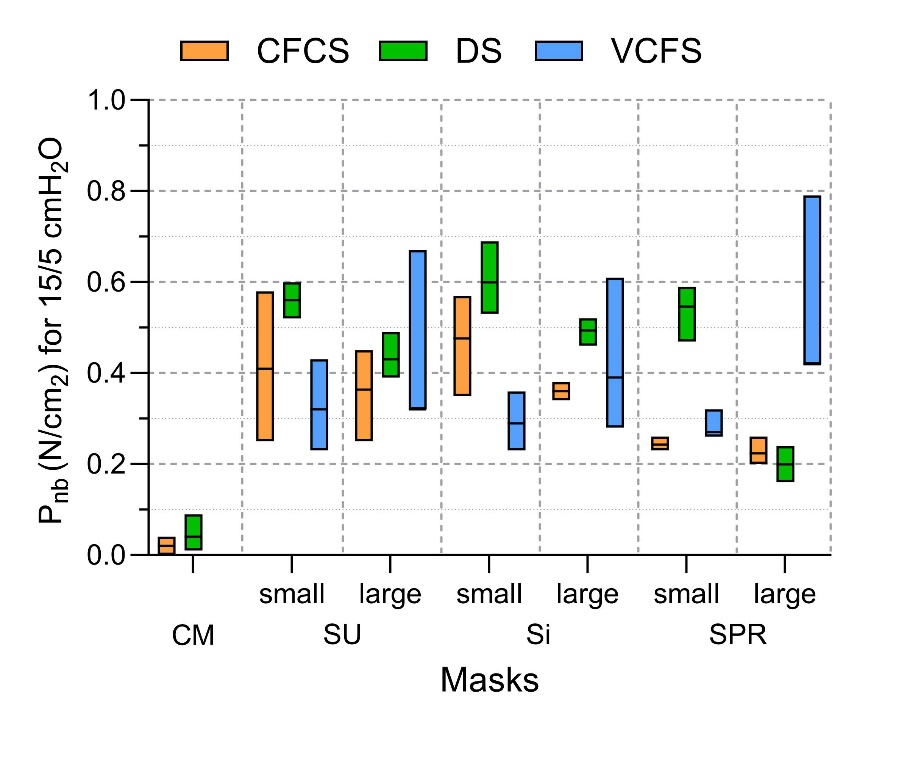


SU


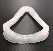


Si


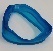


SPR


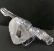


CM


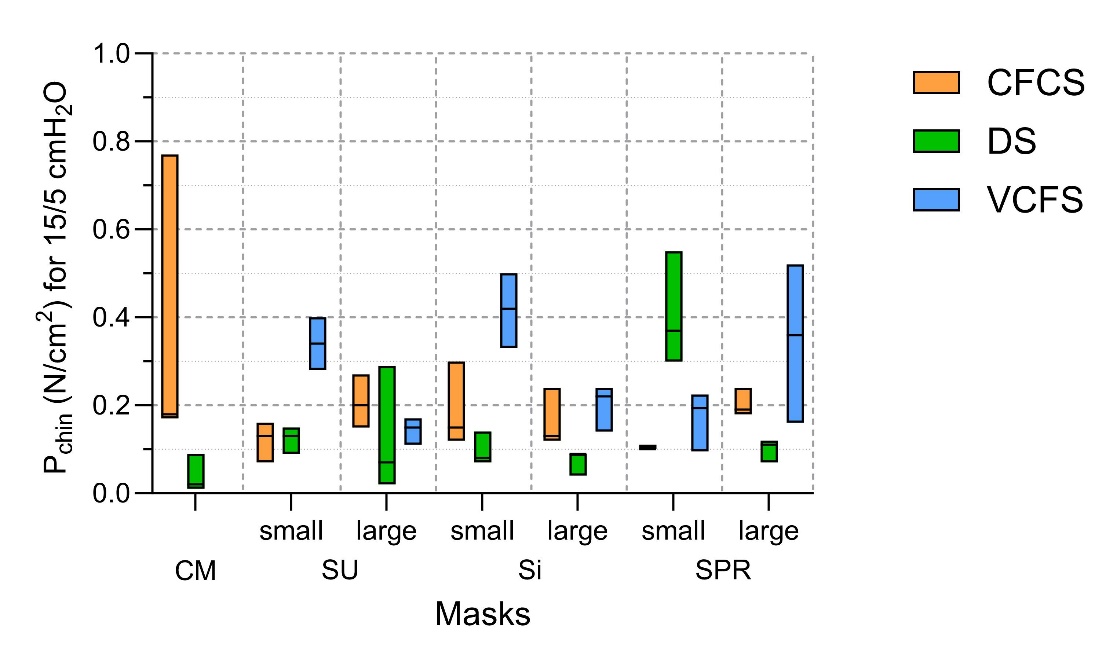

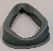

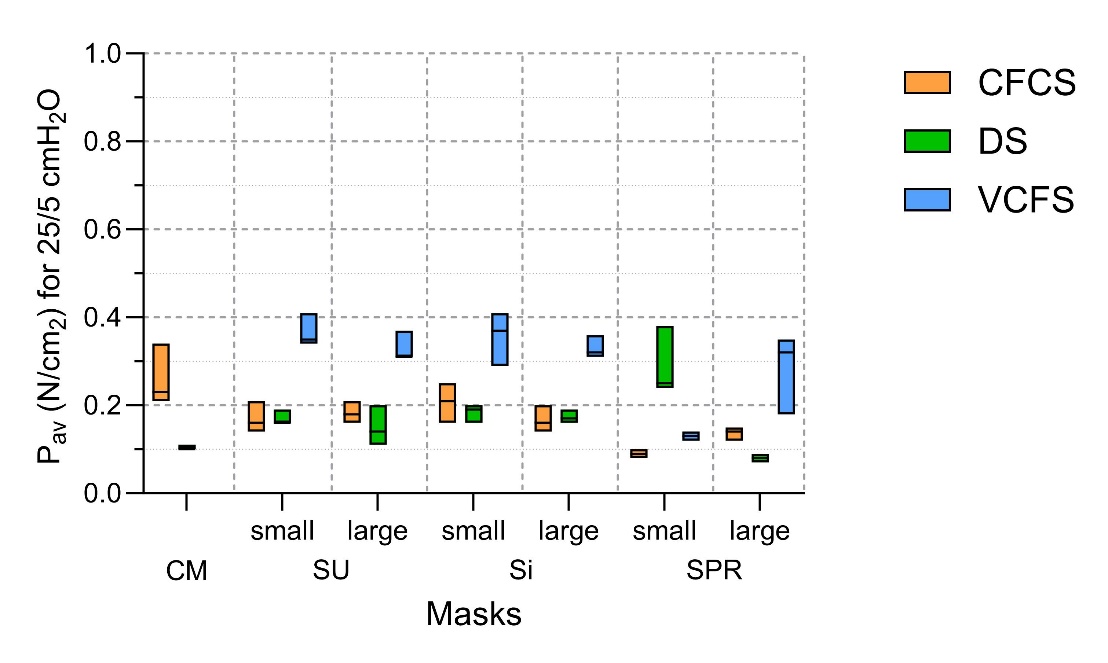

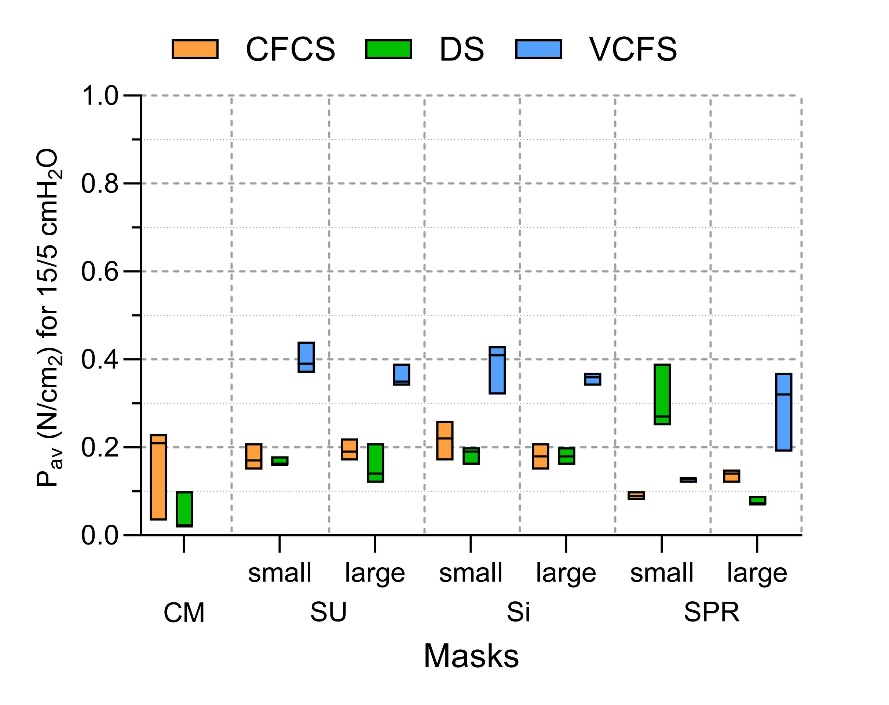

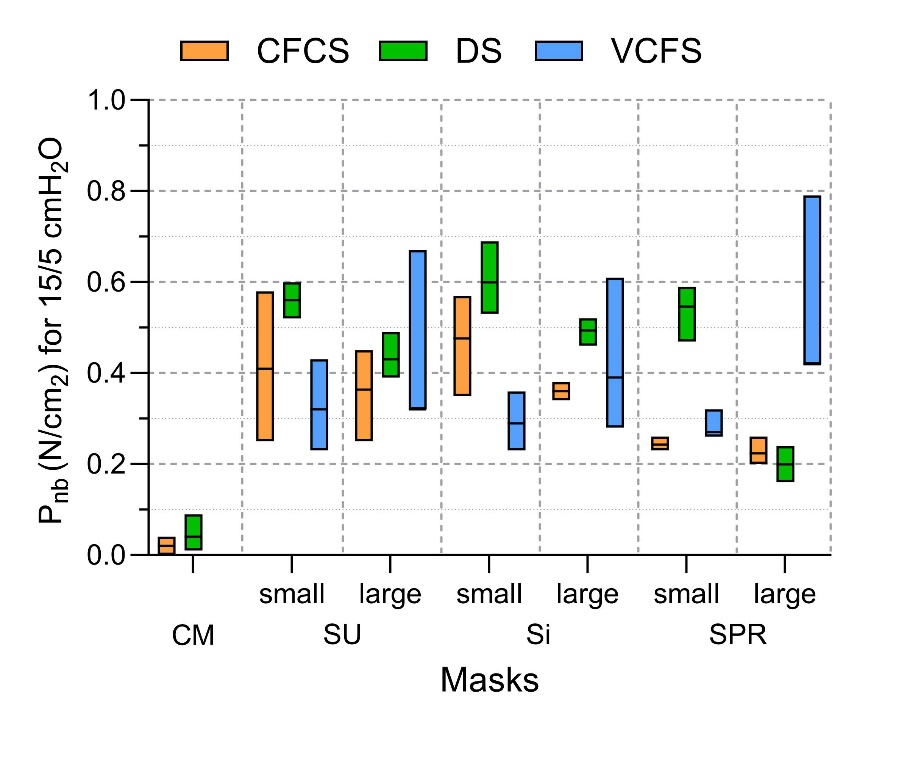


Resulting facial surface pressures (N/cm^2^) (from top to bottom: at the nose bridge sensor (P_nb_); at the chin sensor (P_chin_); average of all six sensors (P_av_) delivered by the commercial mask (CM) and different personalized masks (Silicone Urethane (SU) small and large; Silicone (Si) small and large; and Soft Photopolymer Resin (SPR) small and large) during pediatric non-invasive ventilation bench test simulation in three different test head models (cardiofaciocutaneous syndrome (CFCS), down syndrome (DS) and velocardiofacial syndrome (VCFS). Per facial surface pressure category these data are presented at three different ventilation pressure steps (Peak-Inspiratory Pressure/Positive End-Expiratory Pressure: 15/5 cmH_2_O, 20/5 cmH_2_O and 25/5 cmH_2_O). The boxplots depict median, min and max respectively. The results suggest that there were no major differences in the results obtained from the three different head models.


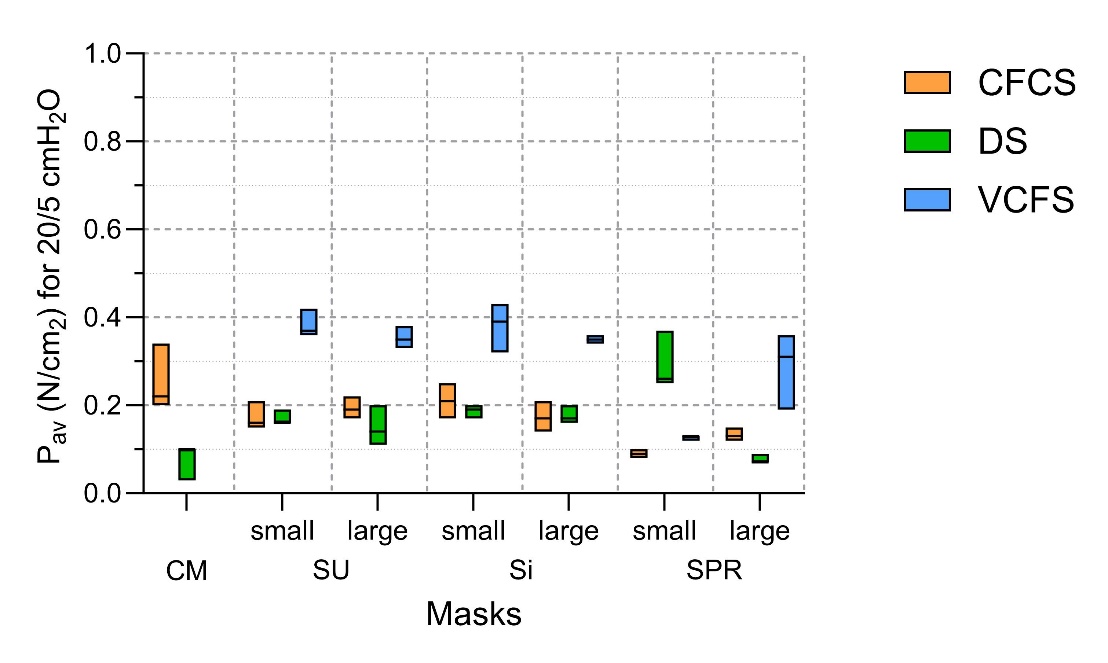

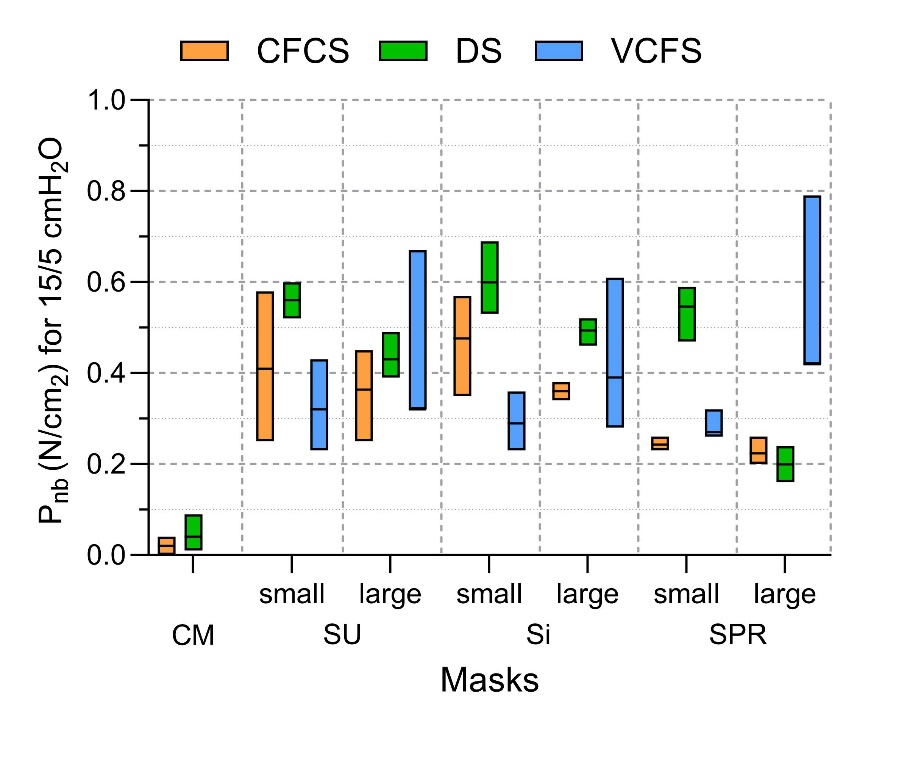


SU


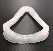


Si


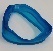


SPR


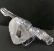


CM


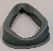

Supplement: Supplementary file 1 — Additional file 1. Supplementary figures. [file 40635_2024_607_MOESM1_ESM.docx]
